# Phosphoinositide-specific phospholipase Cγ1 inhibition induces autophagy in human colon cancer and hepatocellular carcinoma cells

**DOI:** 10.1038/s41598-017-13334-y

**Published:** 2017-10-24

**Authors:** Lianzhi Dai, Xiaolei Chen, Xiaohong Lu, Fen Wang, Yanyan Zhan, Gang Song, Tianhui Hu, Chun Xia, Bing Zhang

**Affiliations:** 1 Medical School, Xiamen University, Xiamen, Fujian, 361102 China; 2 Zhongshan Hospital, Xiamen University, Fujian, 361004 China

## Abstract

Phosphoinositide-specific phospholipase C (PLC) γ1 has been reported to be involved in cancer cell proliferation and metastasis. However, whether PLCγ1 modulates autophagy and the underlying mechanism remains unclear. Here, we investigated the relationship between PLCγ1 and autophagy in the human colon cancer cell line HCT116 and hepatocellular carcinoma cell line HepG2. The results indicated that PLCγ1 inhibition via lentivirus-mediated transduction with shRNA/PLCγ1 or transient transfection with pRK5-PLCγ1 (Y783A) vector increased LC3B-II levels and the number of autophagic vacuoles and decreased p62 levels. Addition of an autophagy inhibitor led to LC3B and p62 accumulation. Furthermore, AMPK activation promoted the autophagy induced by PLCγ1 inhibition by blocking the FAK/PLCγ1 axis. In addition, PLCγ1 inhibition either blocked the mTOR/ULK1 axis or enhanced dissociation of the Beclin1-IP3R-Bcl-2 complex to induce autophagy. Taken together, our findings revealed that PLCγ1 inhibition induced autophagy and the FAK/PLCγ1 axis is a potential downstream effector of the AMPK activation-dependent autophagy signalling cascade. Both blockade of the mTOR/ULK1 axis and dissociation of the Beclin1-IP3R-Bcl-2 complex contributed to the induction of autophagy by PLCγ1 inhibition. Consequently, these findings provide novel insight into autophagy regulation by PLCγ1 in colon cancer and hepatocellular carcinoma cells.

## Introduction

Macroautophagy (hereafter referred to as autophagy) consists of a series of stages; including initiation, elongation and expansion of the phagophore assembly site; formation and maturation of autophagosomes; autophagosome fusion with lysosomes; and digestion^1^. Autophagy can be stimulated by various pathological and physiological states and be dysregulated in several disorders, including cancer. Although studies have presented evidence addressing the relationship between autophagy and tumour progression^1–3^, it is difficult to clearly define the significance of autophagy in the pathological progression of cancer cells. For instance, some studies have illustrated that autophagy suppression promotes tumour progression^4,5^. However, an increase in autophagy can enhance cancer cell aggressiveness and therapy resistance^6,7^. Therefore, investigating the complex regulatory mechanism of autophagy is helpful for understanding the role of autophagy in tumour pathogenesis.

Numerous signalling molecules participate in regulating individual stages in the process, including adenosine 5′-monophosphate (AMP)-activated protein kinase (AMPK), mammalian target of rapamycin (mTOR), unc-51-like autophagy activating kinase 1 (ULK1), Beclin1, Bcl-2, microtubule-associated protein 1 light chain3 (LC3), p62 (also called SQSTM1), AuTophaGy-related genes (ATG) and their respective Atg proteins^1^. Among them, mTOR can phosphorylate ULK1 at S757 to suppress autophagy^8,9^. Beclin1, a component of the Beclin1-Vps34-Vps15 complex, triggers the autophagy protein cascade^10^. LC3 is a major autophagy effector, and the conversion of LC3-I (cytosolic, free form of LC3) to its phosphatidylethanolamine-conjugated and autophagosome membrane-associated form, LC3-II, is an initiating step in autophagy activation in mammals^11^. p62 targets ubiquitinated substrates to autophagosomes via its interaction with LC3B and is required both for formation and degradation of polyubiquitin-containing bodies by autophagy^12^.

Phosphoinositide-specific phospholipase C (PLC) γ1 is activated by both receptor and non-receptor tyrosine kinases and can induce hydrolysis of phosphatidylinositol 4,5-bisphosphate (PIP2) to generate two second messengers, inositol 1,4,5-triphosphate (IP3) and diacylglycerol (DAG), which trigger a series of signalling pathways to regulate cellular processes^13–17^. For instance, depletion of PLCγ expression or inhibition of its activity not only increases cisplatin-induced apoptosis but also suppresses the invasive ability of RhoGDI2-overexpressing SNU-484 gastric cancer cells^15^. PLCγ1 inhibition via cell transduction with lentivirus carrying short hairpin RNA blocked the growth and metastasis of human gastric adenocarcinoma^16^. Therefore, PLCγ has an important role in promoting proliferation and metastasis of cancer cells. However, whether PLCγ is involved in autophagy and the underlying mechanism remains unclear.

Several studies have illustrated a relationship between the two hydrolysis products of PIP2 (IP3 and DAG) induced by PLCγ activity and autophagy. IP3 can activate IP3R to positively or negatively regulate autophagy^18,19^. DAG production is necessary for efficient autophagy of Salmonella, and its localization to bacteria-containing phagosomes precedes antibacterial autophagy^20^. Our previous study also showed that PLCγ1 activated mTOR signalling, which is known to be a negative autophagy regulator, in gastric adenocarcinoma cells^17^. Hence, we considered the possibility that autophagy regulation by PLCγ1 may occur in cancer cells. Both colon cancer and hepatocellular carcinoma are digestive system tumours derived from endoderm and are associated with high mortality. Thus, elucidating their regulatory mechanisms is beneficial for development of cancer therapeutics. Moreover, to date, the regulatory role of PLCγ1 with regard to autophagy in the two types of cancer cells is unclear. In addition, our previous studies of PLCγ1 in gastric carcinoma cells provided some materials and methods for this study. Hence, we investigated the role of PLCγ1 in autophagy in human colon cancer and hepatocellular carcinoma.

In this study, after detecting the expression levels of PLCγ1 and the autophagy marker LC3B in different colon cancer and hepatocellular carcinoma cell lines, we chose the colon cancer cell line HCT116 and hepatocellular carcinoma cell line HepG2 for subsequent experiments. Our results demonstrated that PLCγ inhibition, via either shRNA or transfection with a PLCγ phosphorylation mutant, induced autophagy in HCT116 and HepG2 cells. Furthermore, the focal adhesion kinase (FAK)/PLCγ1 axis was found to be a potential downstream effector of the AMPK activation-dependent autophagy signalling cascade. Finally, we found that blockade of the mTOR/ULK1 axis and dissociation of Beclin1 from the Beclin1-IP3R-Bcl-2 complex contributed to the induction of autophagy by PLCγ1 inhibition. Hence, these findings provide novel insights into autophagy regulation by PLCγ1 in HCT116 and HepG2 cells.

## Results

### Involvement of PLCγ1 in autophagy regulation in HCT116 and HepG2 cells

**Figure 1:**
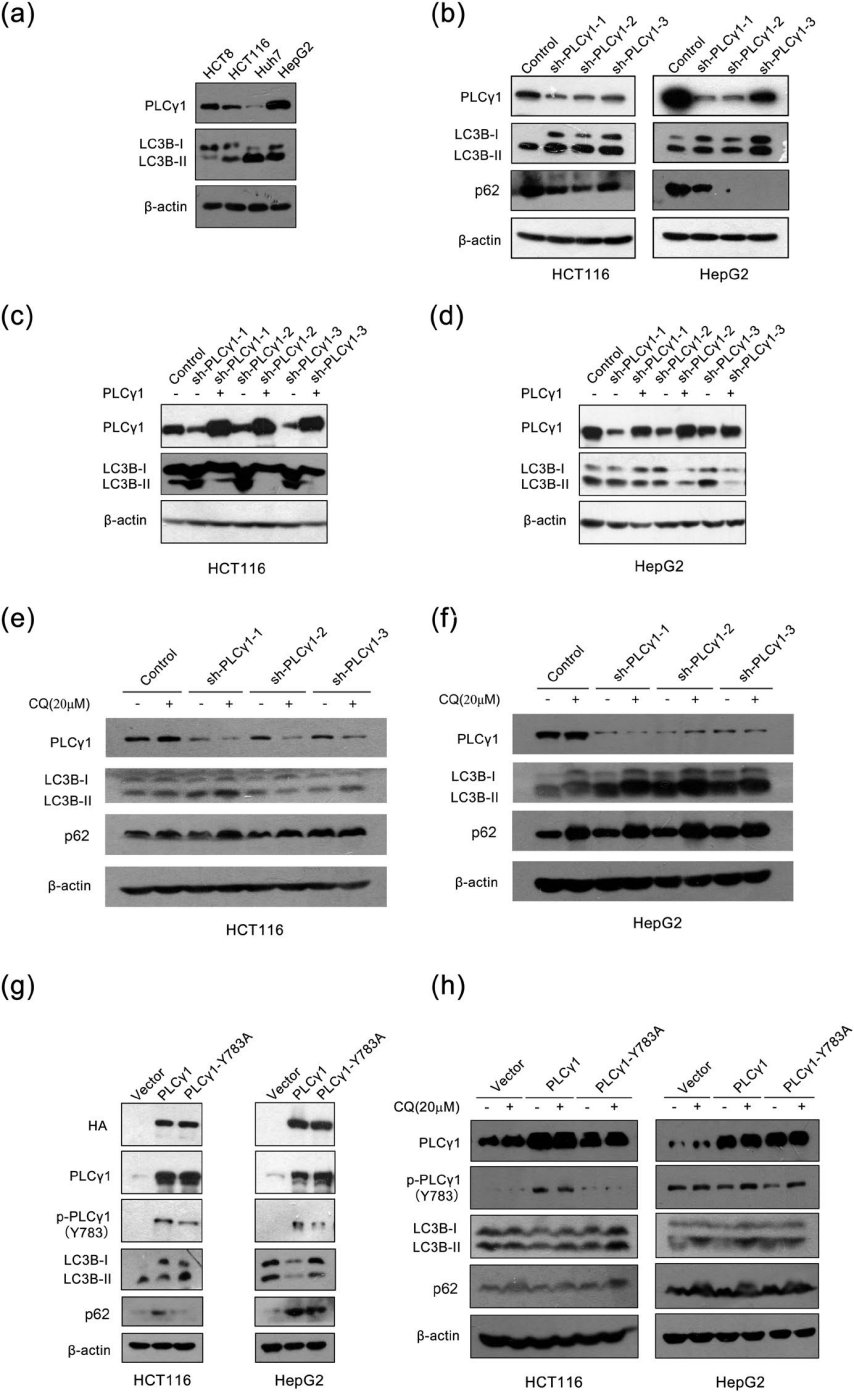
PLCγ1 is involved in autophagy regulation in HCT116 and HepG2 cells. (**a**) HCT8, HCT116, Huh7, and HepG2 cells were cultured, and the PLCγ1, LC3B, and β-actin protein levels were detected in each cell line via western blotting analysis. (**b**) HCT116 and HepG2 cells were transduced with shRNA/PLCγ1-1/2/3 vectors, and the PLCγ1, LC3B, p62, and β-actin protein levels were detected via western blotting. (**c**) and (**d**). HCT116 and HepG2 cells stably expressing shRNA/PLCγ1 were transiently transfected with pRK5-PLCγ1 vectors, and the PLCγ1, LC3B, and β-actin protein levels were detected via western blotting. (**e**) and (**f**) The HCT116 and HepG2 cells transduced with shRNA/PLCγ1-1/2/3 vectors were treated with or without CQ (20 μM) for 24 h, and the PLCγ1, LC3B, p62, and β-actin protein levels were detected via western blotting. (**g**) HCT116 and HepG2 cells were transiently transfected with pRK5-PLCγ1 and pRK5-PLCγ1 (Y783A) vectors, and the HA, PLCγ1, p-PLCγ1, LC3B, p62, and β-actin protein levels were detected with western blotting. (**h**) The transfected HCT116 and HepG2 cells were treated with or without CQ (20 μM) for 24 h, and the PLCγ1, p-PLCγ1, LC3B, p62, and β-actin protein levels were detected with western blotting. The data are representative of three or five independent experiments.

To choose appropriate cell lines for subsequent experiments, we first detected the protein expression levels of PLCγ1 and LC3B (autophagy marker) via western blotting analysis in the colon cancer cell lines HCT116 and HCT8 and hepatocellular carcinoma cell lines HepG2 and Huh7. In the two colon cancer cell lines, lower PLCγ1 and higher LC3B-II expression was observed in HCT116 cells and higher PLCγ1 expression and lower LC3B-II expression was observed in HCT8 cells (Fig. 1(a)). Similarly, in the hepatocellular carcinoma cell lines, we observed higher PLCγ1 expression along with lower LC3B-II expression in HepG2 cells and lower PLCγ1 expression with higher LC3B-II expression in Huh7 cells (Fig. 1(a)). These results indicated that there was a relationship between PLCγ1 and autophagy (LC3B-II expression) in both colon cancer cells and hepatocellular carcinoma cells.

Given that the conversion of LC3B-I to LC3B-II represents an increase in autophagy^1,11^ and that the levels of LC3B (including LC3B-1 and LC3B-II) in HCT116 and HepG2 cells were easier to detect than in HCT8 and Huh7 cells, HCT116 and HepG2 cells were chosen for subsequent experiments. Figure 1(b) shows that PLCγ1 depletion using lentiviral-mediated shRNA/PLCγ1-1/2/3 vectors caused an increase in LC3B-II protein levels. Because the increase in LC3B-II could be attributed to either the induction of early stages of autophagy or inhibition of late stages of autophagic flux^21^, the results not absolutely indicative of autophagy induction. The p62 level, another autophagy marker, was then assessed in HCT116 and HepG2 cells transduced with shRNA/PLCγ1 vectors. As an autophagy substrate, p62 is often degraded when autophagy is induced^12,22^. Hence, the decreased p62 level shown in Fig. 1(b) indicates autophagy induction by shRNA/PLCγ1. Meanwhile, we rescued the effect of shRNA/PLCγ1 on LC3B-II expression by transfecting the two types of cancer cells with pRK5, encoding an HA-tagged PLCγ1 (pRK5-PLCγ1) vector, to overexpress PLCγ1. The results showed that the increased LC3B-II expression induced by shRNA/PLCγ1 was down-regulated by transfection with pRK5-PLCγ1 vectors (Fig. 1(c) and (d)). Furthermore, addition of the autophagy inhibitor chloroquine (CQ) (20 μM) for 24 h, which prevents the fusion of autophagosomes and lysosomes by increasing lysosomal pH, up-regulated the expression levels of LC3B and p62 compared with the untreated group (Fig. 1(e) and (f)). Therefore, shRNA/PLCγ1 was confirmed to induce autophagy in HCT116 and HepG2 cells. In addition, phosphorylation of PLCγ1 at Y783 is essential for its activation^23^, and thus, we then investigated the role of p-PLCγ1 at Y783 in autophagy induction. Figure 1(g) shows that the LC3B-II level increased in cells transfected with pRK5 encoding an HA-tagged PLCγ1 (Y783A) (pRK5-PLCγ1(Y783A)) vector expressing a point mutation at the Y783 site of PLCγ1 compared with cells transfected with pRK5-PLCγ1 vector, while the expression level of p62 decreased. The addition of 20 μM CQ for 24 h enhanced the levels of LC3B and p62 compared with the untreated group (Fig. 1(h)). Hence, inhibition of PLCγ1 phosphorylation also induced autophagy in HCT116 and HepG2 cells. Overall, PLCγ inhibition using either shRNA or transfection with a PLCγ phosphorylation mutant induced autophagy in HCT116 and HepG2 cells.

### Morphological features of autophagy induced by PLCγ1 inhibition in HCT116 and HepG2 cells

**Figure 2:**
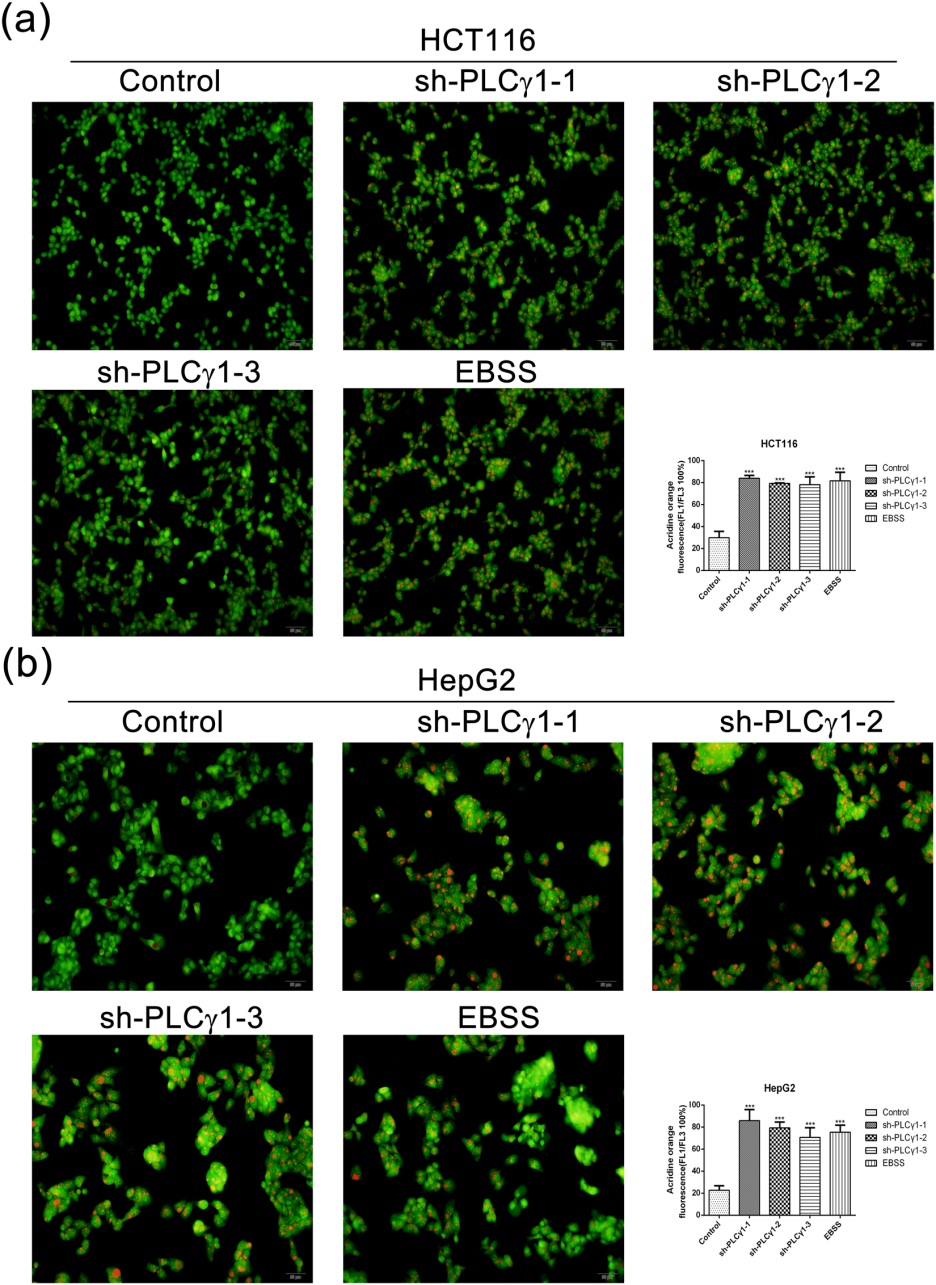
Observation of acidic autophagic vesicles in HCT116 and HepG2 cells under a fluorescence microscope. Cells were transduced with shRNA/PLCγ1 vectors, followed by acridine orange staining. The acidic autophagic vesicles were dyed red and observed under a fluorescence microscope (Magnification ×200, EBSS as the positive control). (**a**) HCT116 cells. (**b**) HepG2 cells. The data are reported as the means ± S.D. of three independent experiments (***P < 0.001 *vs* Control).

**Figure 3:**
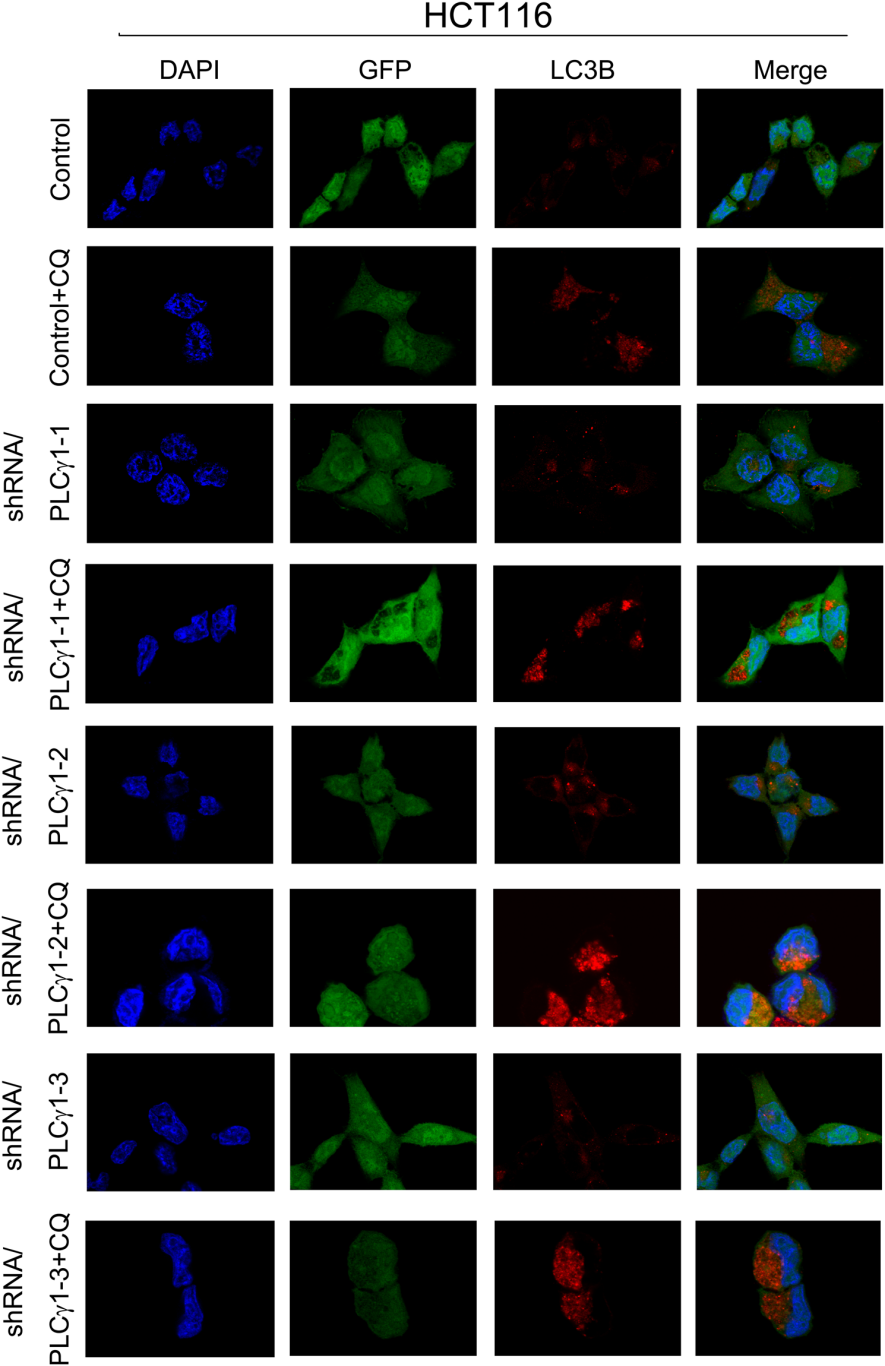
Observation of LC3B puncta in HCT116 cells using laser-scanning confocal microscopy. Cells were transduced with shRNA/PLCγ1 vectors, followed by treatment with or without CQ (20 μM) for 24 h. After immunofluorescence staining was performed, the red immunofluorescence pattern of LC3B was observed under a laser-scanning confocal microscope (magnification ×400).

**Figure 4:**
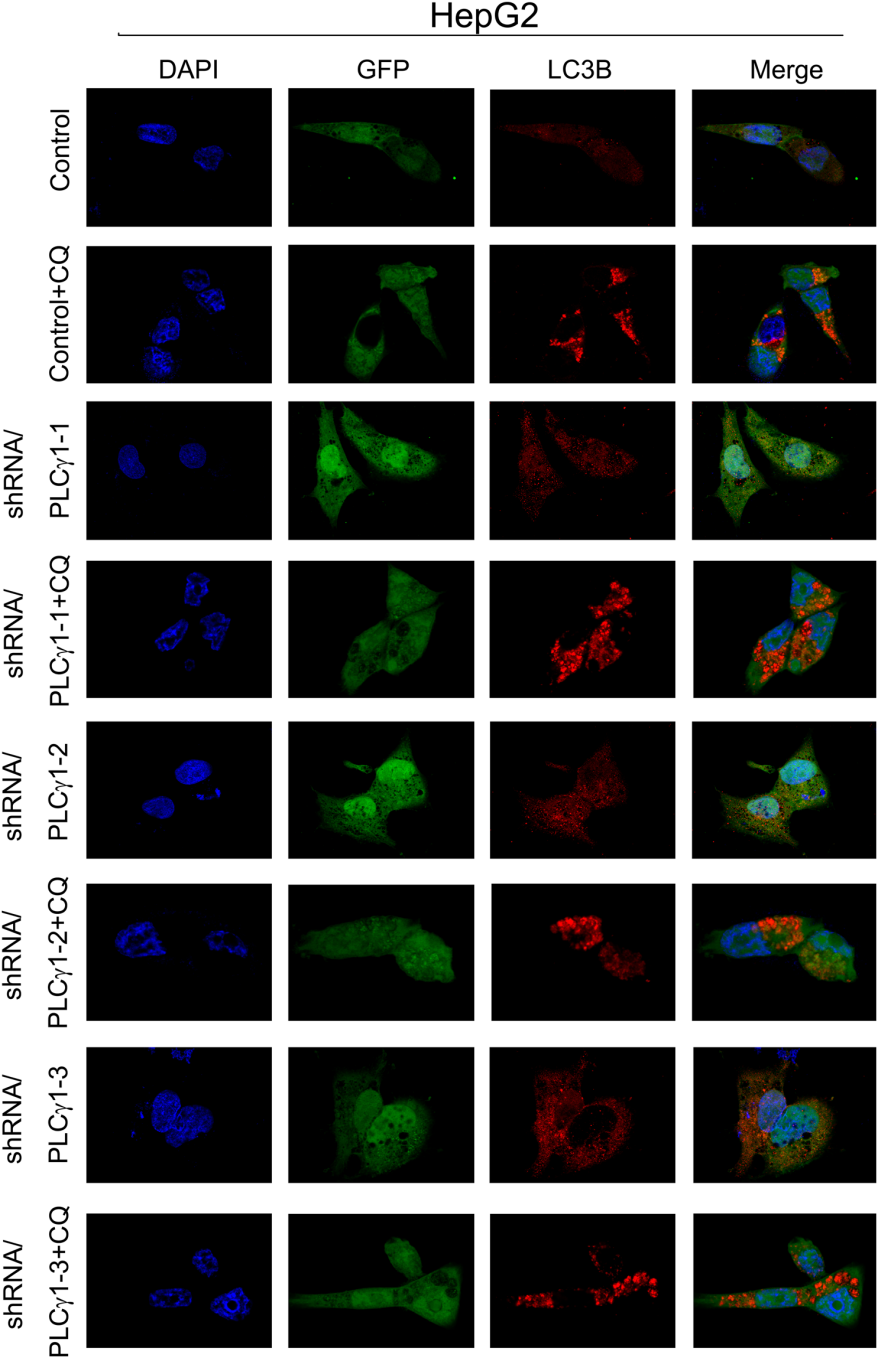
Observation of LC3B puncta in HepG2 cells using laser-scanning confocal microscopy. Cells were transduced with shRNA/PLCγ1 vectors, followed by treatment with or without CQ (20 μM) for 24 h. After the immunofluorescence staining was performed, the red immunofluorescence pattern of LC3B was observed under a laser-scanning confocal microscope (magnification ×400).

**Figure 5:**
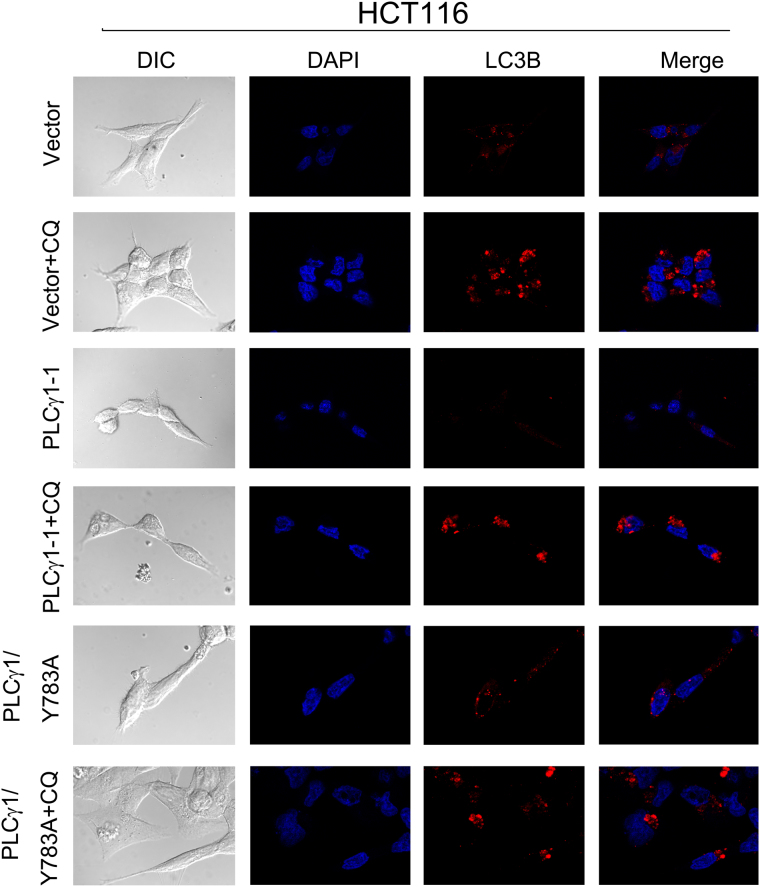
Observation of LC3B puncta in HCT116 cells using laser-scanning confocal microscopy. Cells were transiently transfected with pRK5-PLCγ1 or pRK5-PLCγ1 (Y783A) vector, followed by treatment with or without CQ (20 μM) for 24 h. After the immunofluorescence staining was performed, the red immunofluorescence pattern of LC3B was observed under a laser-scanning confocal microscope (magnification ×400).

**Figure 6:**
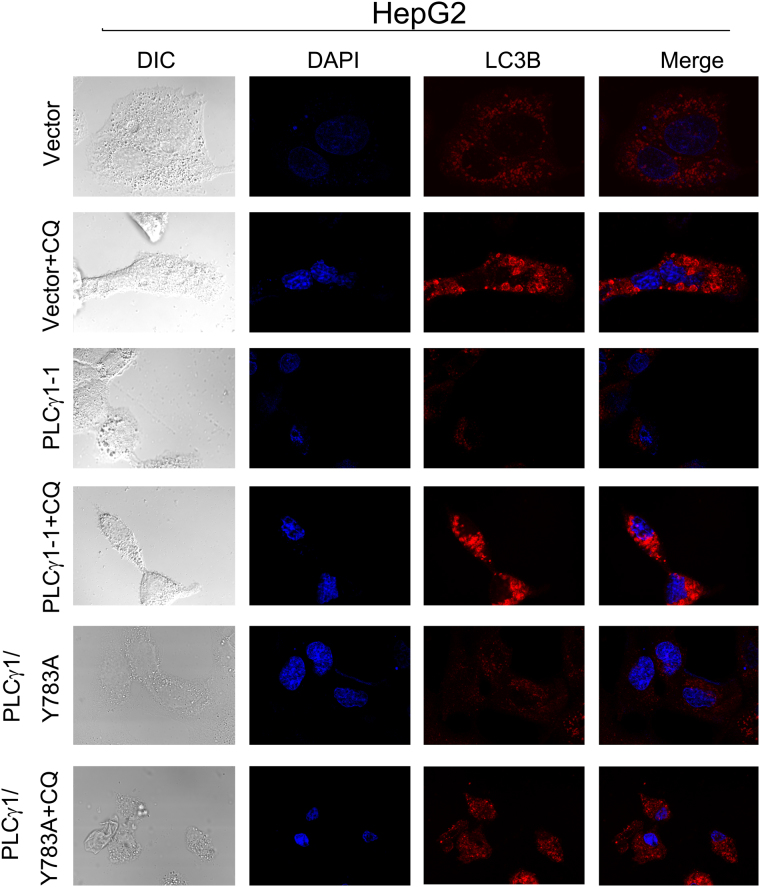
Observation of LC3B puncta in HepG2 cells using laser-scanning confocal microscopy. Cells were transiently transfected with pRK5-PLCγ1 and pRK5-PLCγ1 (Y783A) vectors, followed by treatment with or without CQ (20 μM) for 24 h. After the immunofluorescence staining was performed, the red immunofluorescence pattern of LC3B was observed under a laser-scanning confocal microscope (magnification ×400).

**Figure 7:**
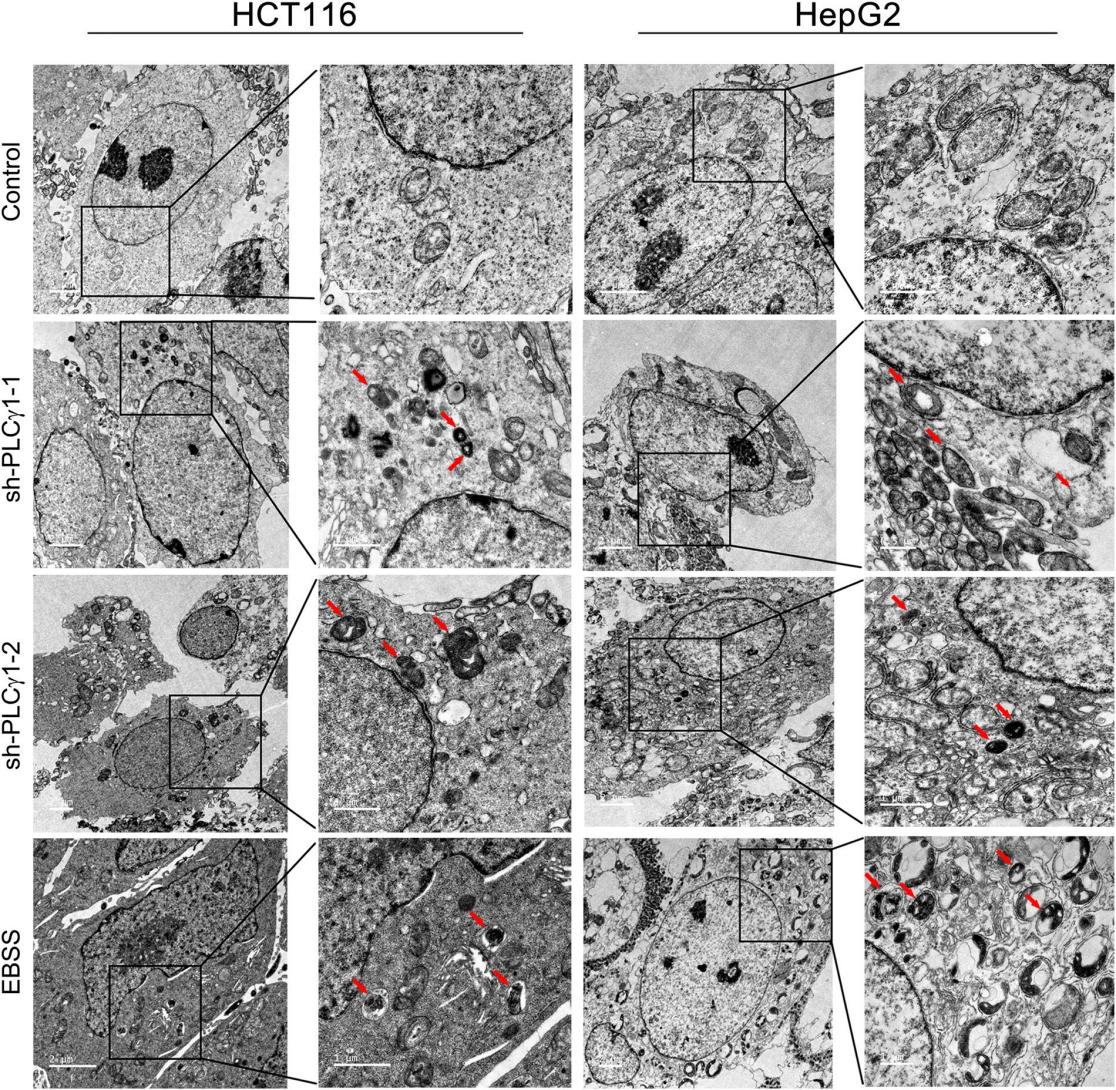
Observation of autophagic vacuoles in HCT116 and HepG2 cells using transmission electron microscopy. Cells were transduced with PLCγ1 shRNA vector, and the autophagic vacuoles (indicated by red arrows, EBSS treatment as the positive control) were observed under a transmission electron microscope.

To further corroborate the occurrence of autophagy, we assessed the morphology of autophagic vacuoles and LC3B puncta structure in HCT116 and HepG2 cells transduced with lentiviral shRNA/PLCγ1 vectors or transfected with pRK5-PLCγ1 or -PLCγ1 (Y783A) vectors using different microscopy techniques. Under a fluorescence microscope, the results of acridine orange staining showed that the fluorescence ratio of acidic vesicles (FL1, red) to nuclei (FL3, green) was significantly higher in cells either transduced with shRNA/PLCγ1-1/2 vectors or treated with starvation medium (Earle’s balanced salt solution, without calcium and magnesium, EBSS; as a positive control) than in control cells (Fig. 2, ***P < 0.001). In agreement with a previous study^24^, the higher ratio represented an increased number of autophagic vacuoles. Under laser-scanning confocal microscopy, a high number of LC3B puncta (dyed red) was observed in cells transduced with shRNA/PLCγ1-1/2/3 vectors compared with control cells; at the same time, the addition of CQ led to LC3B puncta accumulation in these transduced cells (Figs 3 and 4). In addition, the number of LC3B puncta in cells transfected with pRK5-PLCγ1 (Y783A) vector was more than that in cells transfected with pRK5-PLCγ1 vector, and the addition of CQ led to LC3B puncta accumulation in the transfected cells (Figs 5 and 6). At the electron microscopy level, an increased number of vacuole-like structures (indicated by red arrows) were observed in the cytoplasm of cells either transduced with shRNA/PLCγ1-1/2 vectors or treated with EBSS compared with control cells (Fig. 7). The vast majority of the observed vacuoles were surrounded by a single membrane (Fig. 7). These vacuoles were filled with amorphous materials or membranous inclusions or organelles at various stages of degradation, which are the hallmark features of autophagy (autophagosomes or autolysosomes) as described previously^25^. Overall, various morphological features of autophagy were observed in HCT116 and HepG2 cells either transduced with lentiviral-mediated shRNA/PLCγ1 vectors or transfected with pRK5-PLCγ1 (Y783A) vectors, consistent with the results shown in Fig. 1.

### PLCγ1 inhibition induces autophagy by either blocking the mTOR/ULK1 axis or enhancing dissociation of the Beclin1-IP3R-Bcl-2 complex

**Figure 8:**
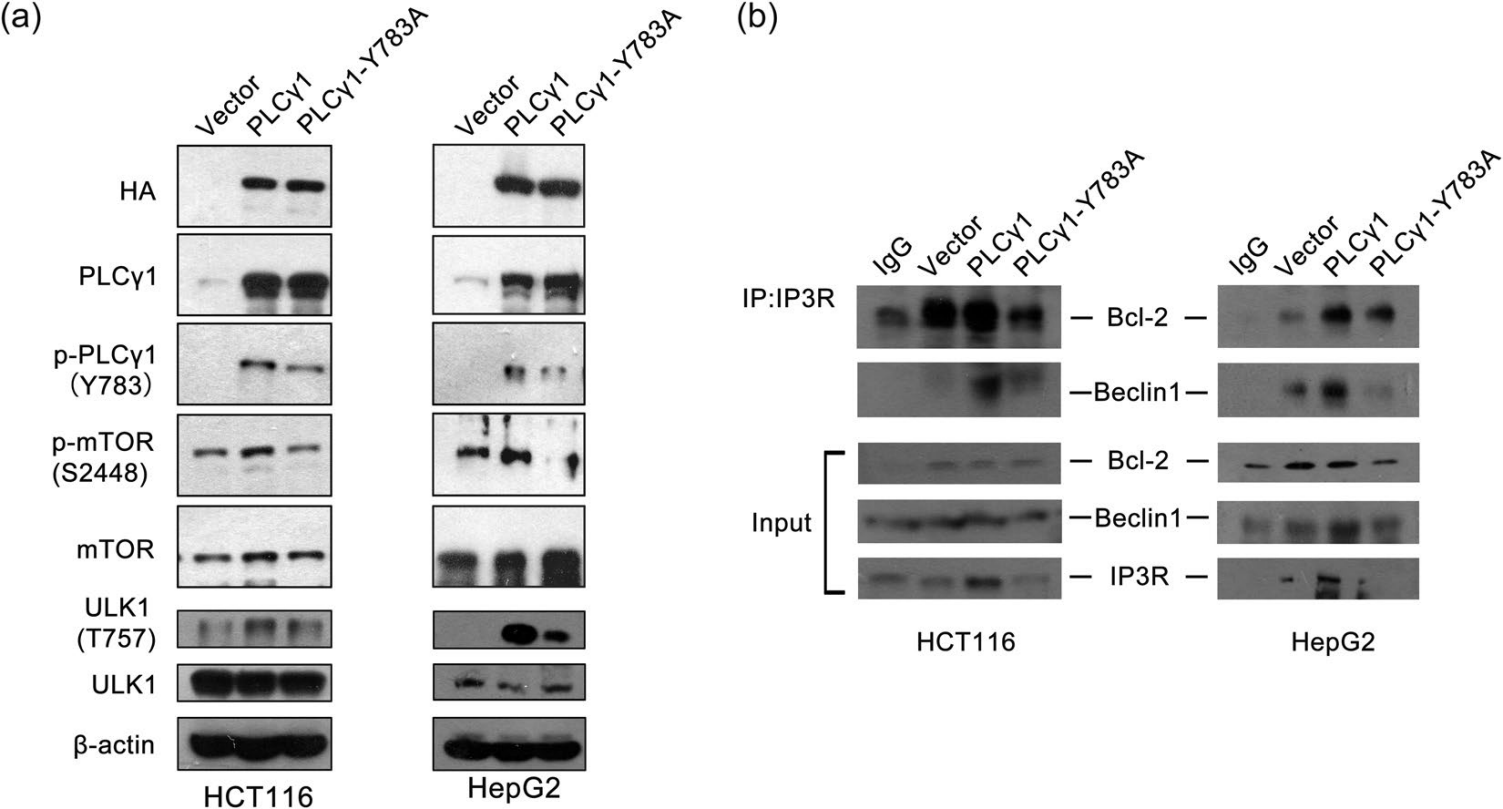
The mTOR/ULK1 axis and Beclin 1-IP3R-Bcl-2 complex are involved in autophagy induction by PLCγ1 inhibition in HCT116 and HepG2 cells. Cells were transiently transfected with pRK5-PLCγ1 and pRK5-PLCγ1 (Y783A) vectors. (**a**) The HA, PLCγ1, p- PLCγ1, mTOR, p-mTOR, ULK1, p-ULK1, and β-actin protein levels were detected with western blotting. (**b**) Protein extracts were subjected to immunoprecipitation with anti-IP3R antibody. The immunoprecipitates were immunoblotted with Beclin1 and Bcl-2 antibodies. The data are representative of three independent experiments.

After the above results demonstrated that PLCγ1 inhibition could induce autophagy in HCT116 and HepG2 cells, it was necessary to determine how PLCγ1 inhibition regulates autophagy. mTOR, an autophagy repressor, has been reported to phosphorylate ULK1 at S757 to suppress autophagy^9^. Our previous study in gastric adenocarcinoma cells also indicated that PLCγ1 could activate mTOR signalling molecules^17^. Thus, we investigated the role of mTOR in PLCγ1 inhibition-induced autophagy. Transfection of cells with pRK5-PLCγ1 (Y783A) led to a decrease in both the p-mTOR and p-ULK1 levels compared with transduction with the pRK5-PLCγ1 vector (Fig. 8(a)). Therefore, the mTOR/ULK1 axis is involved in PLCγ1 inhibition-induced autophagy. On the other hand, IP3, one of the two PIP2 hydrolysis products induced by PLCγ1, diffuses into the cytoplasm and activates IP3R via direct binding. Meanwhile, IP3R can also bind to Beclin1 (through the IP3-binding domain of IP3R) and Bcl-2 (at the middle of the modulatory and transducing domain of IP3R) to form the Beclin1-IP3R-Bcl-2 complex^18^. Dissociation of Beclin1 from the Beclin1-IP3R-Bcl-2 complex can promote autophagy^18,19^, but whether PLCγ1 inhibition induces autophagy by promoting Beclin1-IP3R-Bcl-2 complex dissociation is not known. The results in Fig. 8(b) show that co-immunoprecipitation both between IP3R and Beclin1 and between IP3R and Bcl-2 were weakened in pRK5-PLCγ1 (Y783A) vector-transfected cells compared with pRK5-PLCγ1 vector-transfected cells. Especially, the binding between IP3R and Beclin1 was drastically reduced, indicating that the PLCγ1 (Y783A) mutant enhanced Beclin1 dissociation from the Beclin1-IP3R-Bcl-2 complex (Fig. 8(b)). In sum, the mTOR/ULK1 axis and Beclin1-IP3R-Bcl-2 complex might be involved in the induction of autophagy caused by PLCγ1 inhibition in HCT116 and HepG2 cells.

### The FAK/PLCγ1 axis is a potential downstream effector of AMPK activation-dependent autophagy

PLCγ1 has been reported to be activated by various extracellular factors through receptor or non-receptor tyrosine kinase pathways^13–17^. Hence, understanding the activating signalling network of PLCγ1 is required to elucidate the regulatory mechanism of PLCγ1 inhibition-induced autophagy.

**Figure 9:**
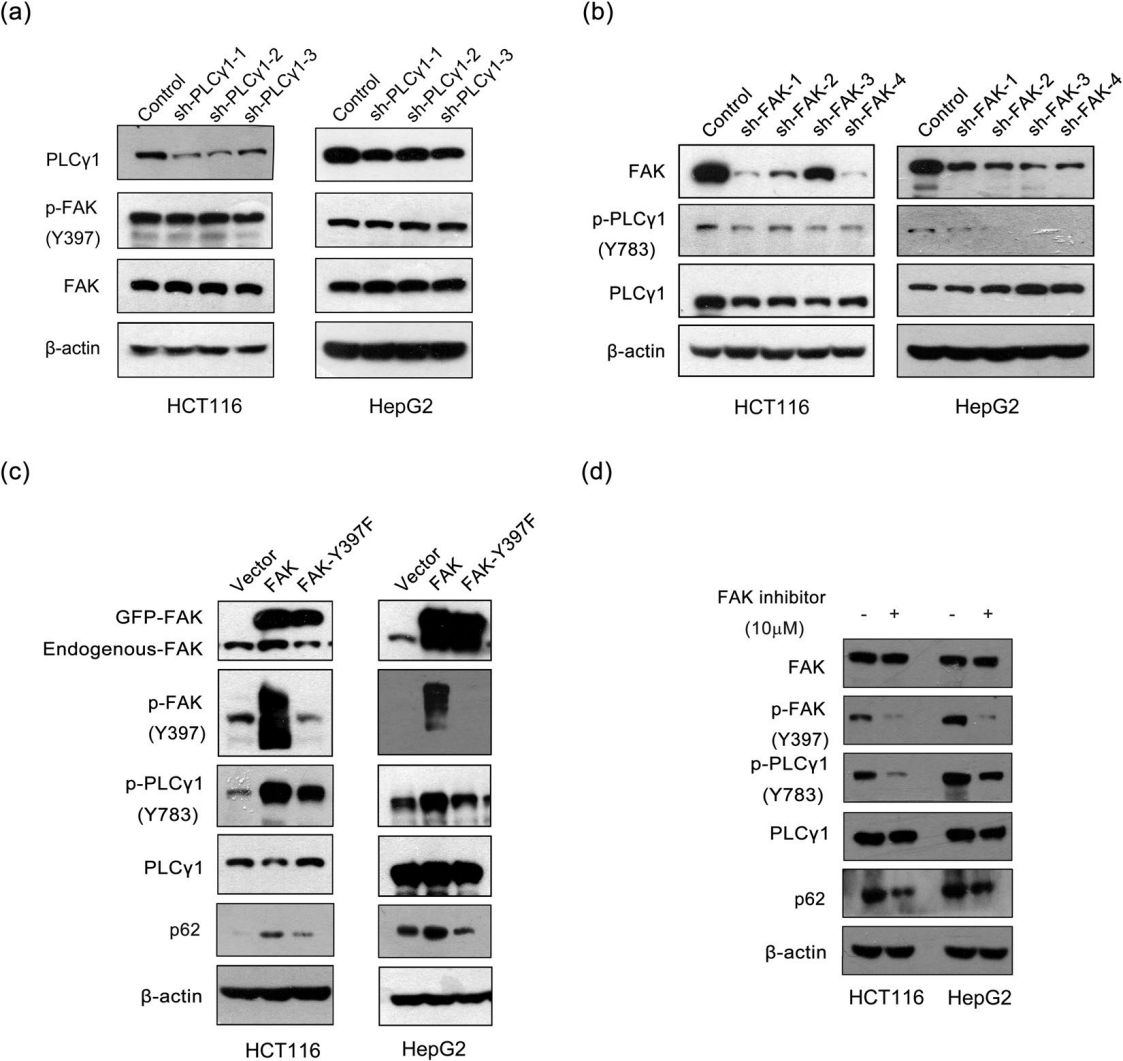
FAK is involved in autophagy induction by PLCγ1 inhibition in HCT116 and HepG2 cells. (**a**) Cells were transduced with shRNA/PLCγ1-1/2/3 vectors, and the PLCγ1, FAK, p-FAK, and β-actin protein levels were detected with western blotting. (**b**) Cells were transduced with shRNA/FAK-1/2/3/4 vectors, and the FAK, PLCγ1, p-PLCγ1, and β-actin protein levels were detected with western blotting. (**c**) Cells were transiently transfected with pEGFP-FAK (Y397F) and pEGFP-FAK vectors, and the FAK, p-FAK, PLCγ1, p-PLCγ1, p62, and β-actin protein levels were then detected with western blotting. (**d**) Cells were treated with a FAK inhibitor 14 (10 μM) for 2 h or 4 h, and the FAK, p-FAK, PLCγ1, p-PLCγ1, p62, and β-actin protein levels were then detected with western blotting. The data are representative of three independent experiments.

FAK has been demonstrated to negatively regulate autophagy^26,27^. Moreover, PLCγ1 can be activated by p-FAK (Y397) in response to integrin-mediated cell adhesion^28^, and thus, determining whether the FAK/PLCγ1 axis was involved in PLCγ1 inhibition-induced autophagy was important. We first investigated whether FAK could activate PLCγ1 in HCT116 and HepG2 cells as reported in a previous study^28^. Figure 9(a) shows that PLCγ1 depletion via transduction with shRNA/PLCγ1-1/2/3 vectors had no effect on FAK expression and phosphorylation. In contrast, FAK depletion via transduction with shRNA/FAK-1/2/3/4 vectors caused a decrease in p-PLCγ1 (Fig. 9(b)). These results indicated that FAK up-regulated PLCγ1 as an upstream regulator. Second, we determined the role of the FAK/PLCγ1 axis in autophagy. The results in Fig. 9(c) show that p-PLCγ1 and p62 levels tremendously decreased in HCT116 and HepG2 cells transfected with pEGFP encoding GFP-tagged FAK (Y397F) (pEGFP-FAK (Y397F)) vector, which contained a point mutation at the Y397 site in FAK, compared with cells transfected with pEGFP encoding GFP-tagged FAK (pEGFP-FAK) vectors expressing wild-type FAK (Fig. 9(c)). Meanwhile, FAK inhibitor 14 (also called Y15), which decreases FAK autophosphorylation at the Y397 site^29^, distinctly reduced the p-PLCγ1 level and decreased the p62 level (Fig. 9(d)). These results revealed that the FAK/PLCγ1 axis might be involved in PLCγ1 inhibition-induced autophagy in HCT116 and HepG2 cells.

**Figure 10:**
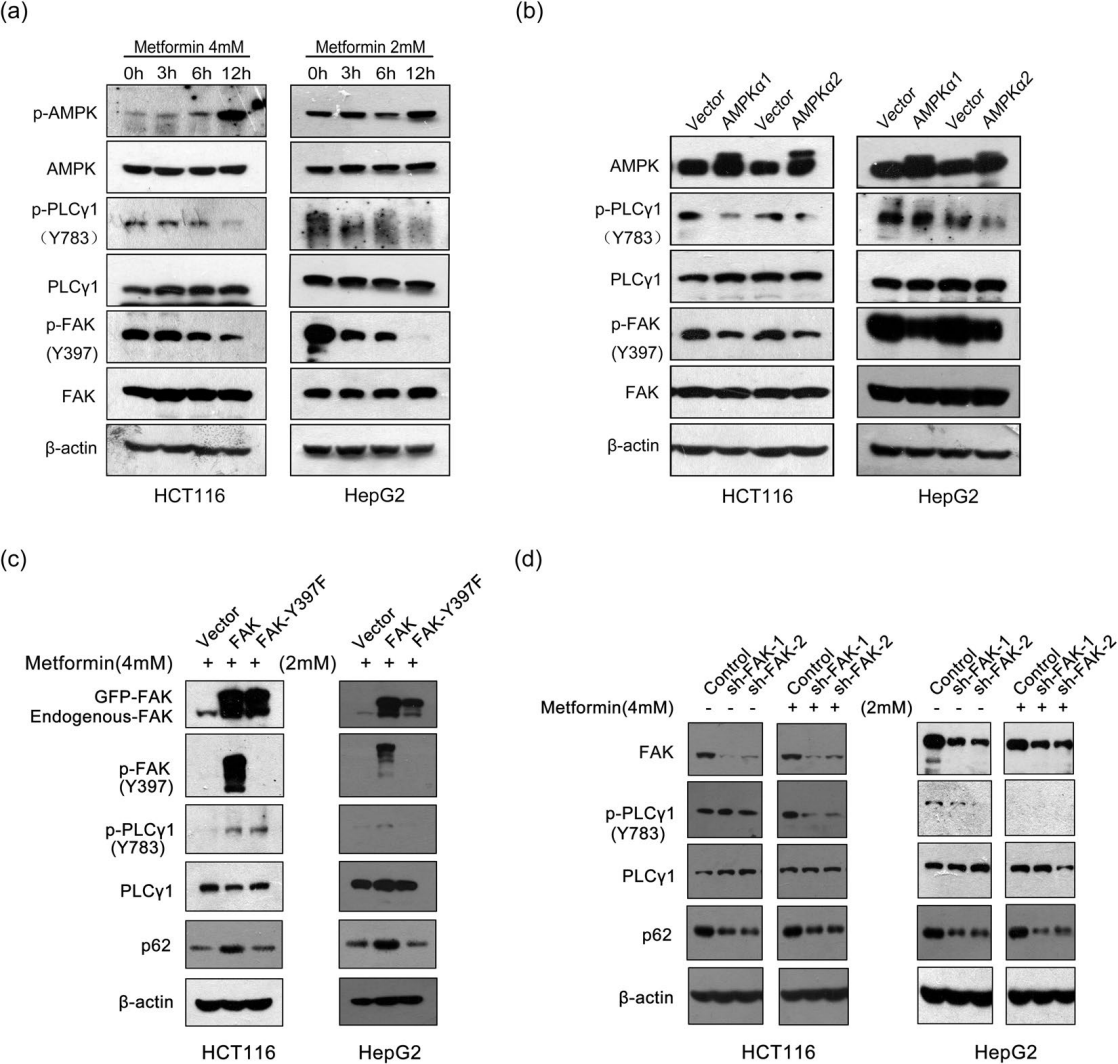
FAK is the key element in AMPK activation to inhibit PLCγ1 in HCT116 and HepG2 cells. (**a**) Cells were treated with metformin (4 mM or 2 mM), an AMPK activator, for 3 h, 6 h, and 12 h. The AMPK, p-AMPK, FAK, p-FAK, PLCγ1, p-PLCγ1, and β-actin protein levels were then detected via western blotting. (**b**) Cells were transiently transfected with AMPKα1 or AMPKα2 vector, and the AMPK, FAK, p-FAK, PLCγ1, p-PLCγ1, and β-actin protein levels were then detected with western blotting. (**c**) Cells were transiently transfected with pEGFP-FAK (Y397F) and pEGFP-FAK vectors, followed by treatment with metformin (4 mM or 2 mM) for 12 h. The FAK, p-FAK, PLCγ1, p-PLCγ1, p62, and β-actin protein levels were then detected with western blotting. (**d**) Cells were transduced with shRNA/FAK-1/2 vectors, followed by treatment with or without metformin (4 mM or 2 mM) for 12 h. The FAK, PLCγ1, p-PLCγ1, p62, and β-actin protein levels were then detected with western blotting. The data are representative of three or five independent experiments.

Activated AMPK is well known to trigger cell autophagy^30,31^, and FAK might be an AMPK substrate^32^. Hence, we investigated whether crosstalk occurs among AMPK, FAK, and PLCγ1 in HCT116 and HepG2 cells. Figure 10(a) shows that both p-PLCγ1 and p-FAK levels decreased with metformin (an AMPK activator) treatment in a concentration-dependent manner^33^. Similarly, transfection with p3xFLAG-CMV10-AMPKα1 (AMPKα1) vector expressing AMPKα1 and p3xFLAG-CMV10-AMPKα2 (AMPKα2) vector expressing AMPKα2 caused an observable decrease in p-PLCγ1 and p-FAK levels (Fig. 10(b)). These results indicated that activated AMPK partially reduced p-FAK and p-PLCγ1 levels in HCT116 and HepG2 cells. Additionally, to unravel whether AMPK might inhibit PLCγ1 via FAK to regulate autophagy, the two types of cells were transiently transfected with different FAK vectors prior to metformin treatment. Compared with the data presented in Fig. 9(c), metformin treatment attenuated the up-regulatory effect of transfection with pEGFP-FAK on PLCγ1 phosphorylation and enhanced the inhibitory effect of transfection with pEGFP-FAK (Y397F) vector on PLCγ1 phosphorylation (Fig. 10(c)). Similar results were observed in cells transduced with shRNA/FAK-1/2 vectors (Fig. 10(d)). These results demonstrated that FAK might be the key element that activates AMPK to inhibit PLCγ1. Meanwhile, compared with the uncontrolled groups, metformin treatment also mitigated the regulatory effect of FAK and FAK (Y397F) vectors on p62 expression (Figs 10(c) and (d)). Therefore, our results indicated that the FAK/PLCγ1 axis could be a potential downstream effector of AMPK activation-dependent autophagy in HCT116 and HepG2 cells.

## Discussion

Our findings showed that PLCγ1 inhibition could elicit autophagy in HCT116 and HepG2 cells. Furthermore, blockade of the mTOR/ULK1 axis and dissociation of Beclin 1 from the Beclin1-IP3R-Bcl-2 complex contributed to the autophagy induced by PLCγ1 inhibition. Additionally, AMPK activation promoted autophagy induction by PLCγ1 inhibition through blocking the FAK/PLCγ1 axis, which is a potential downstream effector of AMPK activation-dependent autophagy. Therefore, PLCγ1 inhibition induces autophagy in HCT116 and HepG2 cells.

Specific LC3B and p62 expression patterns point to different states of autophagy. Very recently, Monique Niklaus *et al*. reported that high expression of both LC3B and p62 was indicative of impaired autophagy activation; in contrast, high LC3B/low p62 expression was indicative of intact autophagy activation^34^. Our results demonstrate that PLCγ1 inhibition via transduction with shRNA/PLCγ1 or transient transfection with PLCγ1 Y783A vector led to an increase in the LC3B-II level and the number of LC3B puncta and vacuole-like structures and a decrease in the p62 level, indicating that autophagy was induced as a result of PLCγ1 inhibition. Moreover, the accumulation of LC3B and p62 induced by CQ are also indicative of autophagy induction through PLCγ1 inhibition. Therefore, our results confirm that PLCγ1 inhibition induces autophagy in HCT116 and HepG2 cells.

The inhibitory function of the mTOR complex 1 in autophagy is well-known^9,35^. For instance, mTOR phosphorylates ULK1 at S757 to suppress autophagy^9^. Moreover, autophagy related to IP3 has been reported to be regulated in an mTOR-related manner in DT40 cells, and DAG negatively regulates mTOR activation in T cells^36,37^. Hence, autophagy induced by PLCγ1 inhibition has been proposed to be associated with mTOR. Our findings that PLCγ1 inhibition led to decreased p-mTOR and p-ULK1 levels support this proposal, suggesting that blockade of the mTOR/ULK1 axis by PLCγ1 inhibition contributed to the autophagy induction in HCT116 and HepG2 cells. Additionally, consistent with the studies of both Vicencio *et al*. and Parys *et al*.^18,19^, we also observed binding of IP3R to Beclin 1 and Bcl-2 in HCT116 and HepG2 cells. Our findings further showed that the binding between IP3R and Beclin1 was weakened in pRK5-PLCγ1 (Y783A) vector-transfected cells, indicating that PLCγ1 inhibition promoted dissociation of the Beclin1-IP3R-Bcl-2 complex, resulting in Beclin 1 release to trigger the autophagy protein cascade. Therefore, we suggest that blockade of the mTOR/ULK1 axis and dissociation of Beclin1 from the IP3R-Beclin1-Bcl-2 complex contributed to the autophagy induced by PLCγ1 inhibition in HCT116 and HepG2 cells.

FAK autophosphorylation at Y397 creates a binding site for many SH2 domain-containing molecules, including PLCγ1, promoting their activities^38,39^. Our results also demonstrated that both depletion of FAK and the point mutation at the Y397 site of FAK reduced the p-PLCγ1 level in HCT116 and HepG2 cells. Thus, FAK activated PLCγ1 in HCT116 and HepG2 cells. Some studies have reported that FAK negatively regulates autophagy^26,27^. For instance, FAK-regulated signalling controls Src-selective autophagy^26^. Activation of FAK by Salmonella suppresses autophagy and promotes bacterial survival in macrophages^27^. Consistent with these studies, our findings that both FAK Y397A and shRNA/FAK caused a decrease in the p62 level demonstrated the inhibitory effect of FAK on autophagy in HCT116 and HepG2 cells. Therefore, blockade of the FAK/PLCγ1 axis might be involved in the induction of autophagy by PLCγ1 in HCT116 and HepG2 cells.

Mounting evidence suggests that increased AMPK activity induces autophagy by regulating other signalling molecules^9,40,41^. LXRXX(S/T) is a consensus amino acid sequence for AMPK-mediated phosphorylation of substrate proteins, and FAK has the AMPK substrate sequence (LNRREES), suggesting that FAK might be an AMPK substrate^32^. Moreover, recent studies have demonstrated that AMPK-dependent phosphorylation of ULK1 mediates phosphorylation and activation of FIP200, leading to release of FAK from inhibition by the autophagy initiator FIP200, which in turn inhibits FAK-directed tumour cell motility and ultimately cancer cell metastasis^42,43^. Thus, FAK has been confirmed as a potential AMPK substrate involved in AMPK-dependent autophagy. On the other hand, activated AMPK inhibited p-PLCγ1 in bone marrow-derived mast cells^44^. These studies determined that both FAK and PLCγ1 could be inhibited by AMPK. Consistent with the above studies, we observed that both FAK and PLCγ1 were negatively regulated by AMPK. In addition, our findings demonstrated that FAK was the key element in AMPK activation to inhibit PLCγ1 in this context and that both FAK and PLCγ1 were involved in AMPK activation-dependent autophagy. Hence, the FAK/PLCγ1 axis is a potential downstream effector of AMPK activation-dependent autophagy, and activated AMPK might trigger the autophagy induced by PLCγ1 inhibition via blockade of the FAK/PLCγ1 axis in HCT116 and HepG2 cells.

**Figure 11:**
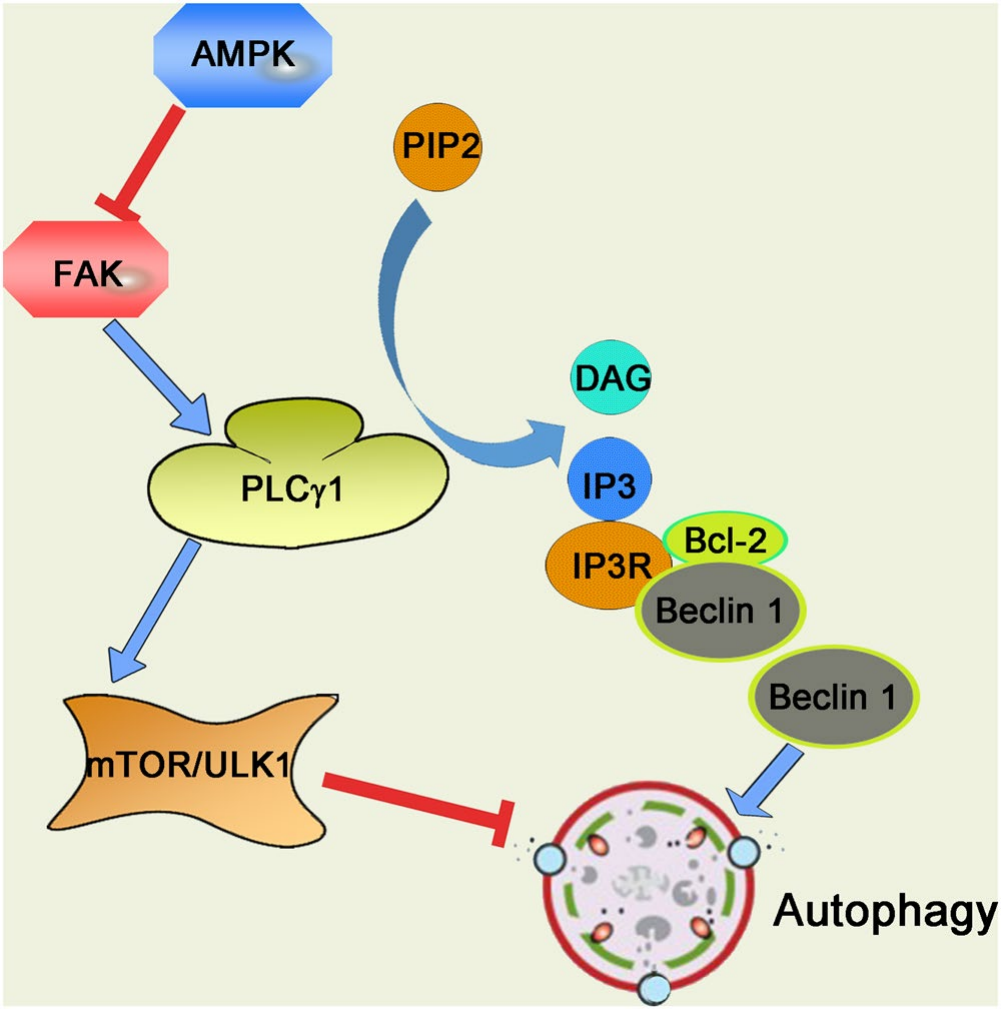
Model of how PLCγ1 inhibition induces autophagy in human colon cancer and hepatocellular carcinoma cells.

In conclusion, our findings reveal a novel and important role of PLCγ1 in regulating autophagy and demonstrate that PLCγ1 inhibition induces autophagy via blockade of the mTOR/ULK1 axis and reduced binding between IP3R and Beclin1. The FAK/PLCγ1 axis might be a potential downstream effector of AMPK activation-dependent autophagy. Consequently, activated AMPK inhibited the FAK/PLCγ1 axis to block the mTOR/ULK1 axis or dissociate the Beclin1-IP3R-Bcl-2 complex, triggering the autophagy protein cascade in HCT116 and HepG2 cells (Fig. 11).

## Materials and Methods

### Cell culture

Human colon cancer HCT116 and HCT-8 cells, human hepatocellular carcinoma HepG2 and Huh7 cells, and HEK293T cells were obtained from the Shanghai Institute of Cell Biology, Chinese Academy of Sciences (Shanghai, China). HCT116 and HCT-8 cells were maintained in McCoy’s 5A (Sigma-Aldrich, Shanghai, China) and RPMI1640 (Gibco) medium, respectively. The other cell lines were cultured in DMEM (Gibco). The media were supplemented with 10% foetal bovine serum (FBS), 100 U/mL penicillin, and 100 μg/mL streptomycin, and the cells were cultured at 37 °C in a water-saturated atmosphere of 5% CO_2_.

### Reagents and antibodies

Antibodies against PLCγ1, p-PLCγ1 (Tyr783), AMPK, p-AMPK (T172), LC3B, mTOR, p-mTOR (Ser2448), FAK, p-FAK (Y397), p-ULK1 (S757), Bcl-2, and Beclin1 were purchased from Cell Signaling Technology Inc. (Beverly, MA, USA). Antibodies targeting IP3R, ULK1, and p62 were purchased from Abcam Inc. (Cambridge, MA, USA). Anti-β-actin peroxidase antibody and the HA-probe were purchased from Sigma-Aldrich in China (Shanghai, China) and Santa Cruz Biotechnology (Santa Cruz, CA, USA), respectively. Other reagents were of the highest grade commercially available.

### Plasmid construction and transfection

Short hairpin RNA (shRNA) targeting both PLCγ1 (sh-PLCγ1) and FAK (sh-FAK) was purchased from Gene Chem (Gene Chem, Shanghai, China) (Table 1). After HCT116 and HepG2 cells were transfected with the different sh-PLCγ1 and sh-FAK vectors using a lentiviral transfection strategy, stable cell lines were obtained under the pressure of puromycin (2 μg/mL, BioVision, Inc., Milpitas, CA, USA). In addition, HCT116 and HepG2 cells were transiently transfected for 48 h with pRK5-HA- PLCγ1 (pRK5-PLCγ1) vector expressing PLCγ1 and pRK5-HA-PLCγ1 (Y783A), (pRK5-PLCγ1 (Y783A)) vector expressing a point-mutant at the Y783 site of PLCγ1^45^, p3xFLAG-CMV10-AMPKα1 (AMPKα1) vector expressing AMPKα1, p3xFLAG-CMV10-AMPKα2 (AMPKα2) vector expressing AMPKα2^46^, pEGFP-FAK vector expressing FAK, or pEGFP-FAK (Y397F) vector expressing a point-mutant at the Y397 site of FAK (Addgene plasmids 50515 and 50516) using Lipofectamine 2000 or Lipofectamine 3000 according to the manufacturer’s procedure (Invitrogen, Carlsbad, CA, USA). The expression of PLCγ1, p-PLCγ1, FAK, p-FAK, AMPK, and p-AMPK was detected with western blotting analysis prior to the other experiments.

**Table 1:** Primers of Sh-RNAs.

Gene name	Primer sequences	
PLCG1.OMIM*172420	Sh1	5′CcgggcCATTGACATTCGTGAAATTctc gagAATTTCACGAATGTCAATGgcTTTTTg3′.
	Sh2	5′CcggccAGATCAGTAACCCTGAATTctcgagAATTCAG GGTTACTGATCTggTTTTTg3′
	Sh3	5′CcggccTGTGAACCACGAATGGTATctcgagATACCATTCGTGGTTCAC AggTTTTTg3′
PTK2 OMIM*005607	Sh1	5′CcgggcCCAGGTTTACTGAACTTAActcgagTTAAGTTCAGTAAACCTGGgcTTTTTg3′
	Sh2	5′CcggccGATTGGAAACCAACATATActcgagTATATGTTGGTTTCCAATCggTTTTTg3′
	Sh3	5′CcggctTGGCCCTGAGGACATTATTctcgagAATAATGTCCTCAGGGCCAagTTTTTg3′
	Sh4	5′CcggccGGTCGAATGATAAGGTGTActcgagTACACCTTATCATTCGACCggTTTTTg3′

### Real-time PCR (RT-PCR)

After total RNA in different cancer cells was extracted using TRIzol (Invitrogen, CA, USA), cDNA was synthesized with 1 µg of total RNA at 37 °C for 15 min using a Primescript RT Master Mix Kit (Takara, Dalian, China). Real-time PCR was then performed using an ABI StepOnePlus Sequence Detection System v2.1 (Applied Biosystems, Singapore) with a SYBR Premix Ex Taq II Kit (Takara, Dalian, China). As described in previous studies^16,47^, the results were normalized to GAPDH and analysed using SDS software v2.1. The primers used for quantitative PCR to measure gene expression levels are listed in Table 2.

**Table 2:** Primers in quantitative PCR.

Gene name	Primer sequence (5′-3′)
GAPDHOMIM *138400	Forward 5′-GGAAGGTGAAGGTCGGAGTCA-3′Reverse 5′-GTCATTGATGGCAACAATATCCACT-3′
MAP1LC3BOMIM *609604	Forward 5′-ATACAAGGGAAGTGGCTATC-3′Reverse 5′-TTACACTGACAATTTCATCC-3′
PLCG1OMIM*172420	Forward 5′-TGTCCCACAGACCAACGC-3′Reverse 5′-ATTCCGCTTCCGCACCAG-3′

### Western blotting analysis

Protein extracts were subjected to SDS-PAGE (6–12%) and transferred to a PVDF membrane (GE Healthcare, Hertfordshire, UK) as described previously^16,17^. The membrane was incubated with various antibodies as required at 4 °C overnight, followed by the addition of the corresponding secondary antibodies at room temperature for 1 to 2 h. An enhanced chemiluminescence (ECL) detection kit was used to detect antibody reactivity (Pierce, Rockford, IL, USA).

### Immunoprecipitation assay

Protein extracts were lysed, and 400 μg of protein was mixed with 8 μl of Protein A&G Sepharose (Sigma-Aldrich, Shanghai, China) and 8 μl of anti-IP3R antibody or immunoglobulin (IgG) control for 3 h at 4 °C. Immunoprecipitation immunoblotting of the sample was then performed using anti-IP3R, anti-Beclin1, and anti-Bcl-2 antibodies as described previously^18,48^.

### Immunofluorescence assay

For staining endogenous LC3B, according to previous studies^18,20^, cells were fixed in 4% paraformaldehyde, permeabilized with 0.1% Triton X-100 for 30 min, blocked with 5% BSA and incubated with anti-LC3B antibody overnight, followed by incubation with Cy3-conjugated secondary antibody (Boster, Wuhan, China) for 1 h in the dark. Nuclei were counterstained with 4′,6-diamidino-2-phenylindole (DAPI, 50 mg/ml, Sigma) for 1 min. The stained cells were finally visualized under a fluorescence microscope (Leica Tcs Sp2 SE, Leica, Shanghai, China).

### Acridine orange staining

Cells were plated in 12-well plates, washed with PBS, and stained with 1 μM acridine orange (Sigma-Aldrich, 318337) for 15 min at 37 °C as described previously^24,49,50^.The acidic vesicular organelles (autophagic vacuoles) were then observed under an inverted fluorescence microscope (Olympus, IX51, Japan) as orange/red fluorescent cytoplasmic vesicles, while the nuclei were stained green. The mean red:green fluorescence ratio indicated changes in autophagic vacuoles. The mean fluorescence intensity of acridine orange staining was calculated with IPP 10.0 software.

### Transmission electron microscopy

Based on typical sample preparation procedures for transmission electron microscopy^51^, cells were scraped and then pelleted by centrifugation at 2000 × g for 15 min at 4 °C, followed by fixation for 2 h at 4 °C in 2.5% glutaraldehyde in 0.1 M PBS (PH7.4). According to the standard procedure, samples were dehydrated and embedded in Embed-812 resin. Then, 70-nm sections were cut using an ultramicrotome (Leica EM UC7, LEICA, Shanghai, China) and stained with uranyl acetate and lead citrate. Finally, autophagosomes were observed with a transmission electron microscope (Tecnai G2 Spirit BioTWIN, FEI Company, Hillsboro, Oregon, USA).

### Statistical analysis

Differences between the groups were examined for statistical significance using Student’s t-test with GraphPad Prism 5 software (GraphPad Software, Inc., La Jolla, CA, USA). A value of P < 0.05 was considered significant.

## Electronic supplementary material

supplementary materials
