# Supplementary material for: Phosphoinositide-specific phospholipase Cγ1 inhibition induces autophagy in human colon cancer and hepatocellular carcinoma cells
Source: Sci Rep. 2017 Oct 24;7:13912. doi: 10.1038/s41598-017-13334-y (PMC5654964; doi:10.1038/s41598-017-13334-y)

Phosphoinositide-specific phospholipase Cγ1 inhibition induces autophagy in human colon cancer and hepatocellular carcinoma cells

Lianzhi Dai[1,#,] , Xiaolei Chen[2,#] , Xiaohong Lu[1], Fen Wang[1], Yanyan Zhan[1], Gang Song[1], Tianhui Hu[1] , Chun Xia[2,*], Bing Zhang[1,*]

[1] Medical School, Xiamen University, Xiamen, Fujian, 361102, China

[2]Zhongshan Hospital, Xiamen University, Fujian, 361004, China

[#]These authors contributed equally to this work.

Emails: Lianzhi Dai(598688513@qq.con); Xiaolei Chen(1282172084@qq.com); Xiaohong Lu(532495891@qq.com); Fen Wang(731522119@qq.com);Yanyan Zhan(yyzhan@xmu.edu.cn); Gang Song(gangsongsd@xmu.edu.cn); Tianhui Hu(thu@xmu.edu.cn)

*Correspondence and requests for materials should be addressed to Chun Xia (chunxia@xmu.edu.cn) or Bing Zhang (cristal66@xmu.edu.cn)

Figure 1A

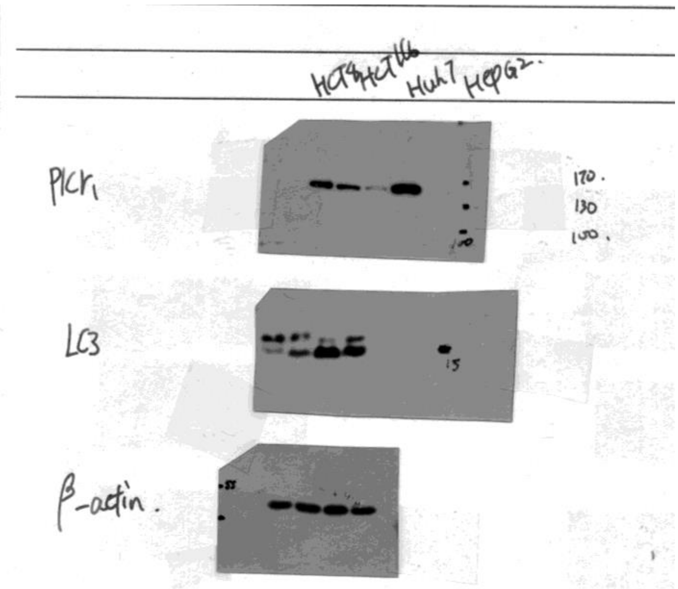

Figure1B(HCT116&HepG2)

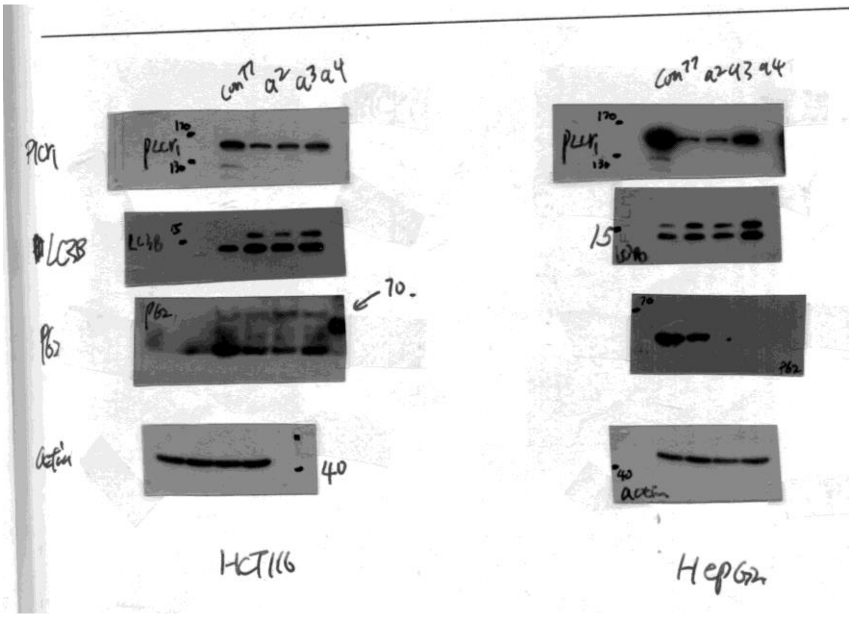

Figure1C(HCT116)&1D(HepG2)

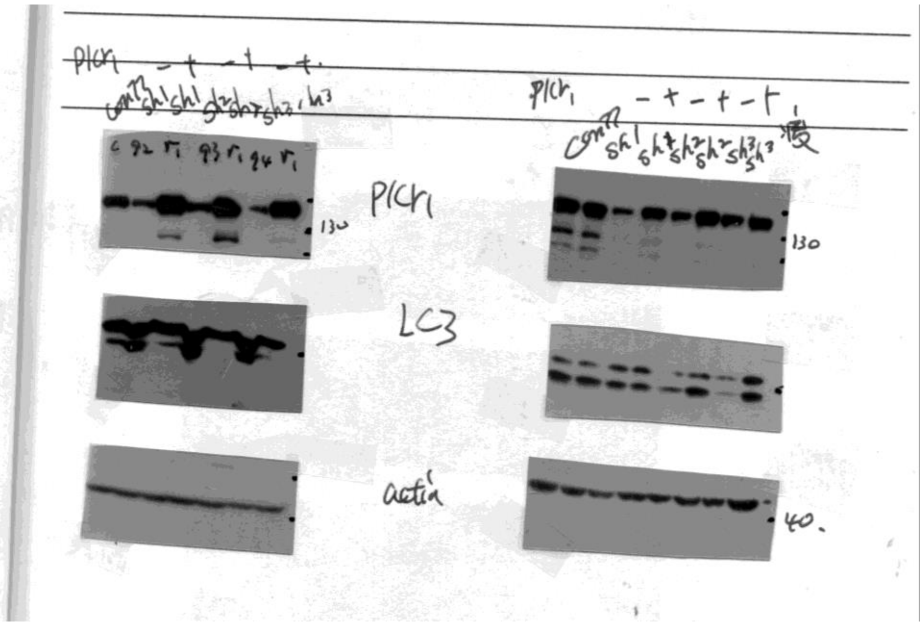

Figure1E(HCT116)

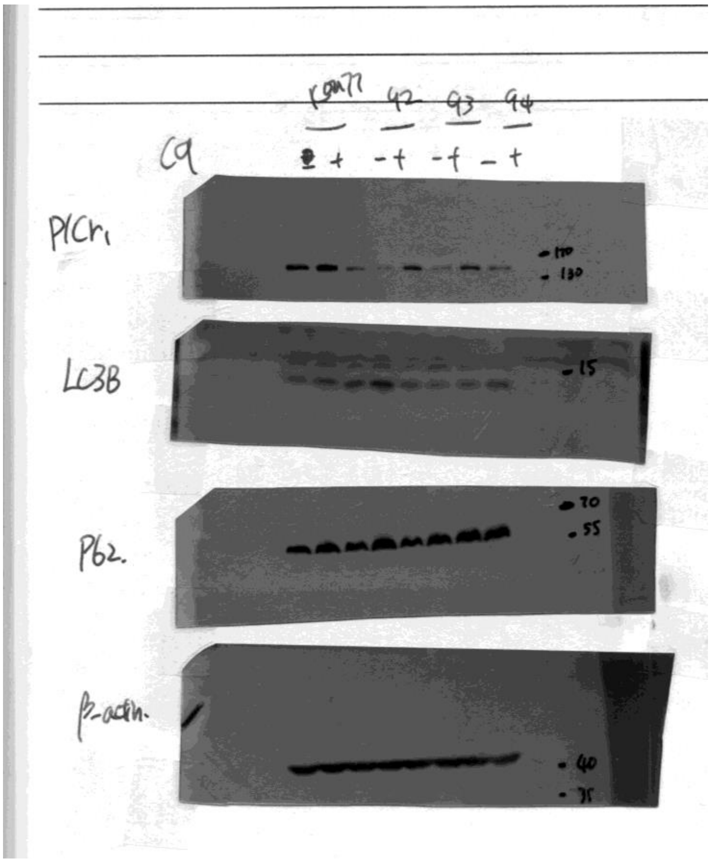

Figure1F(HepG2)

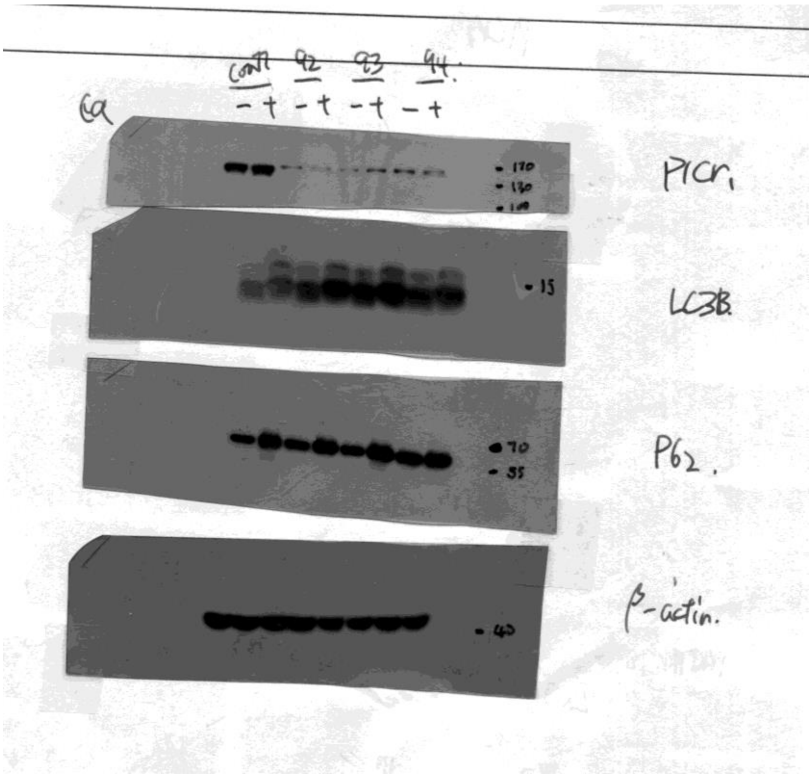

Figure1G-1&Figure8A-1(HCT116)

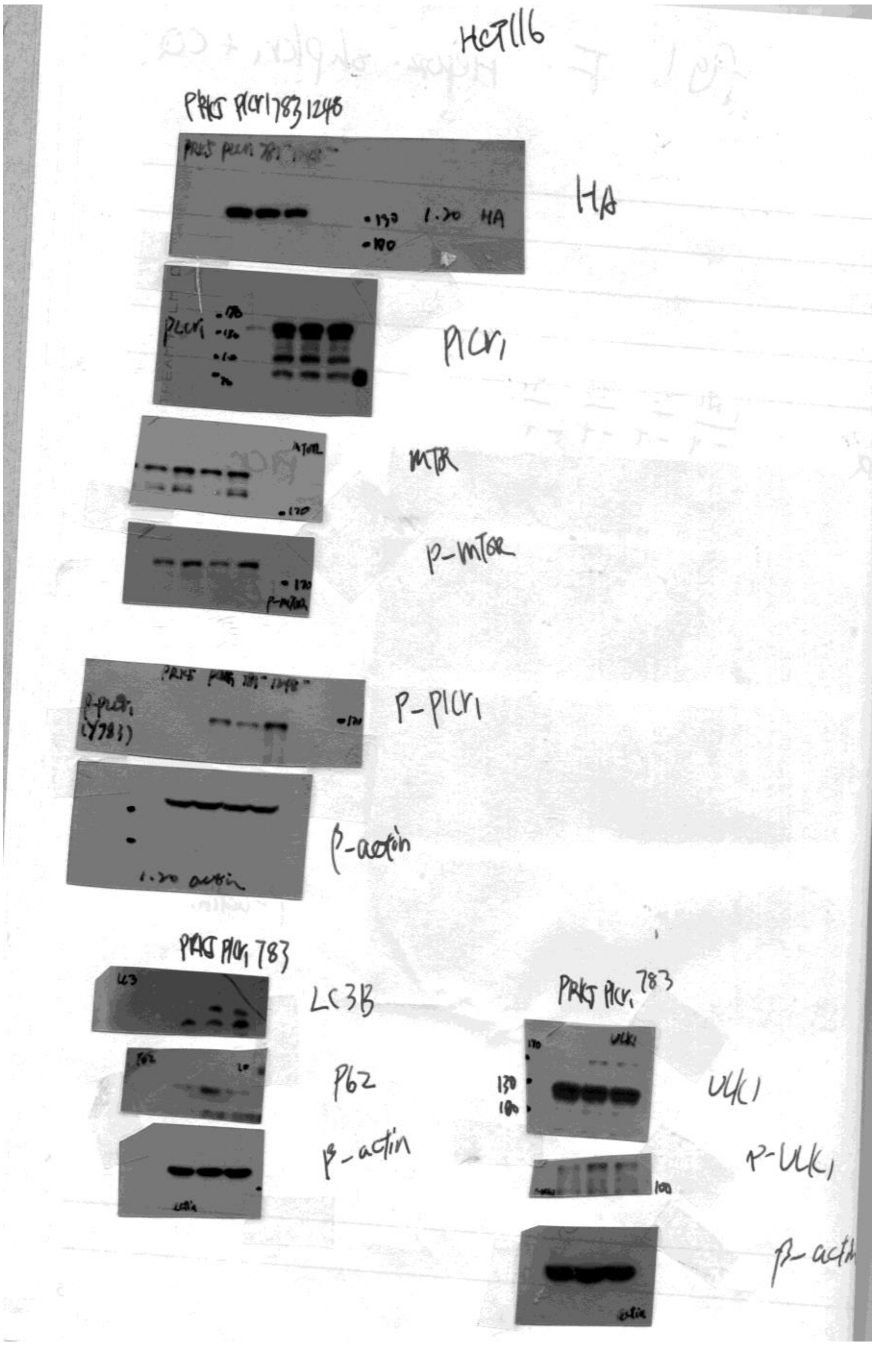

Figure1G-2&Figure8A-2(HepG2)

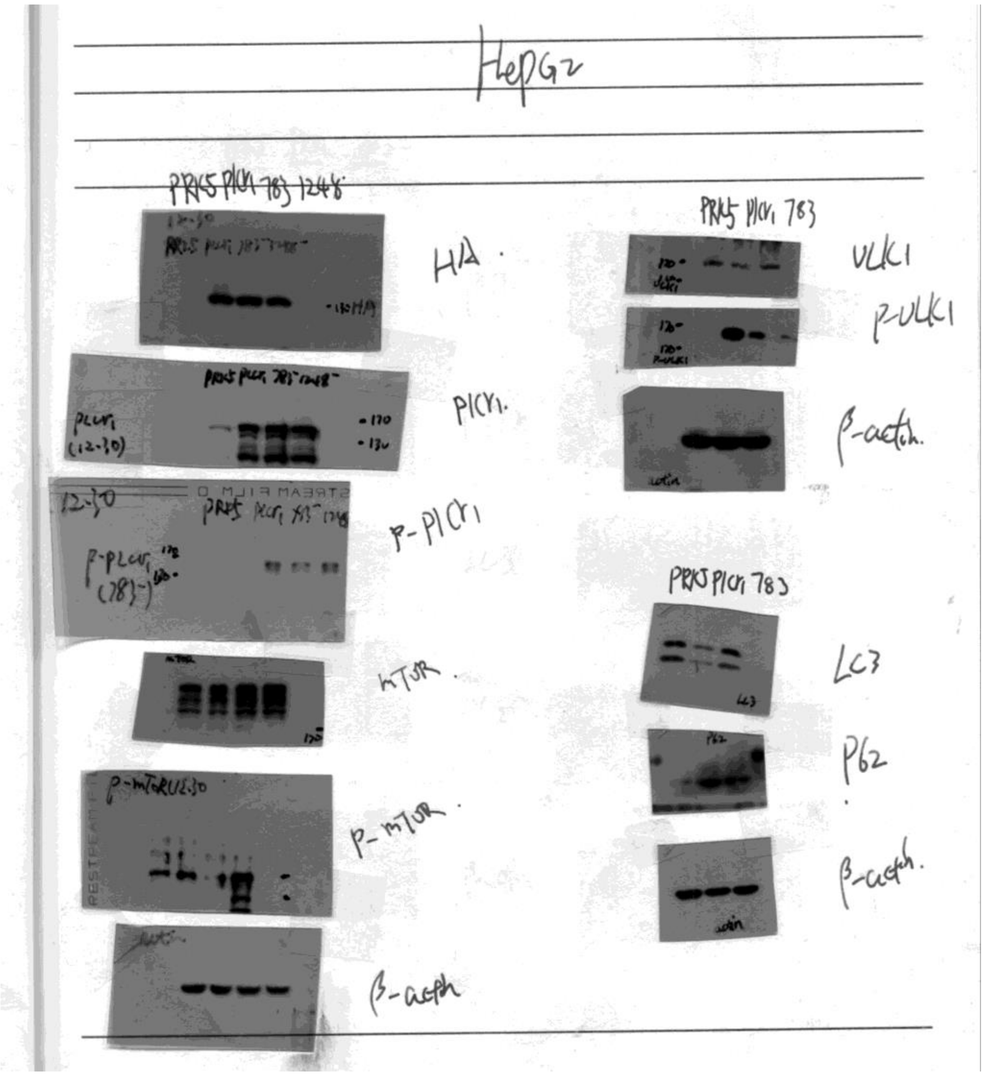

Figure1H(HCT116&HepG2)

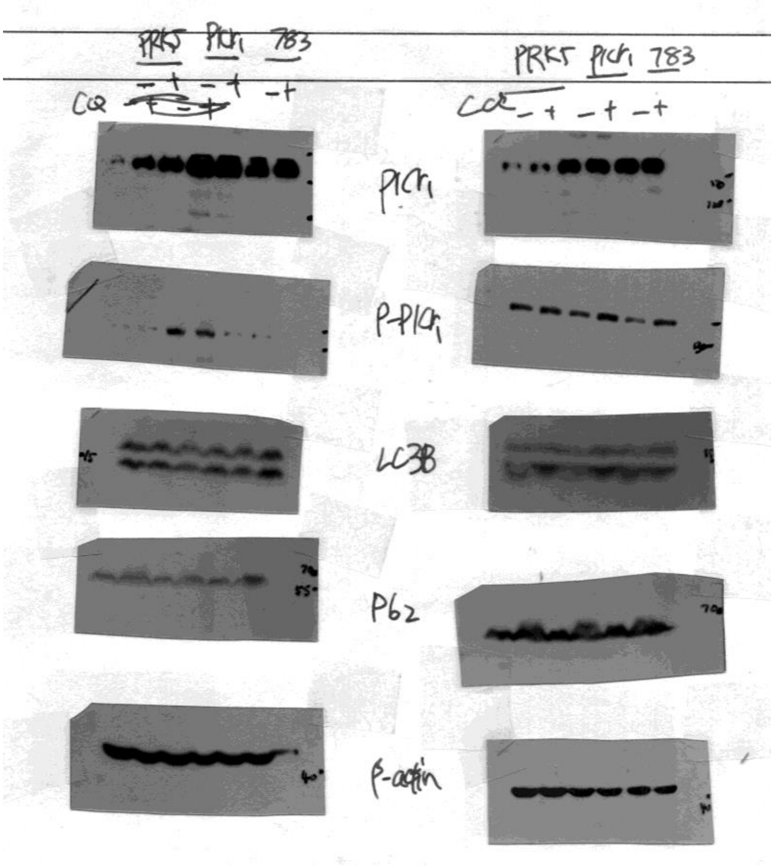

Figure8B(HCT116&HepG2)

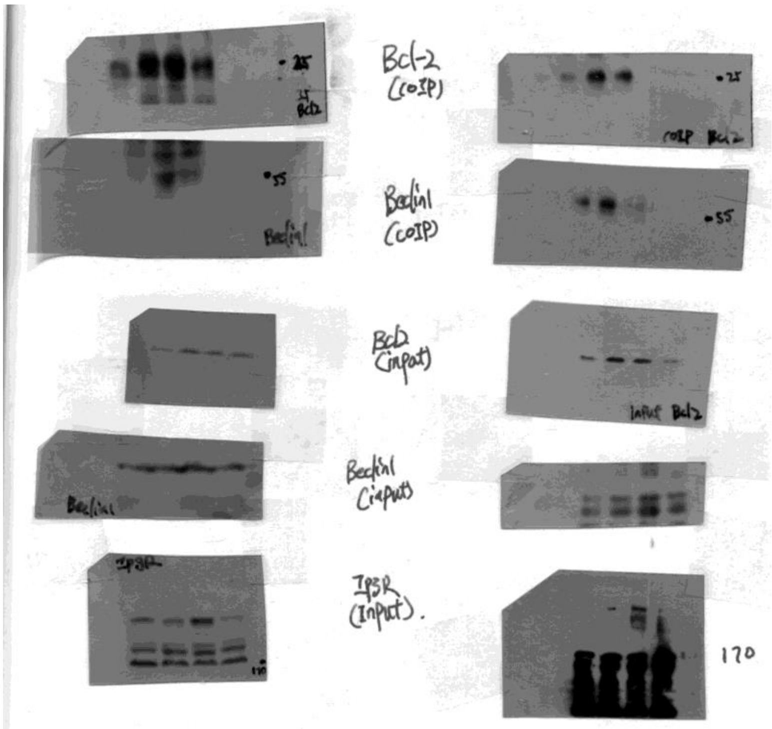

Figure9A(HCT116&HepG2)

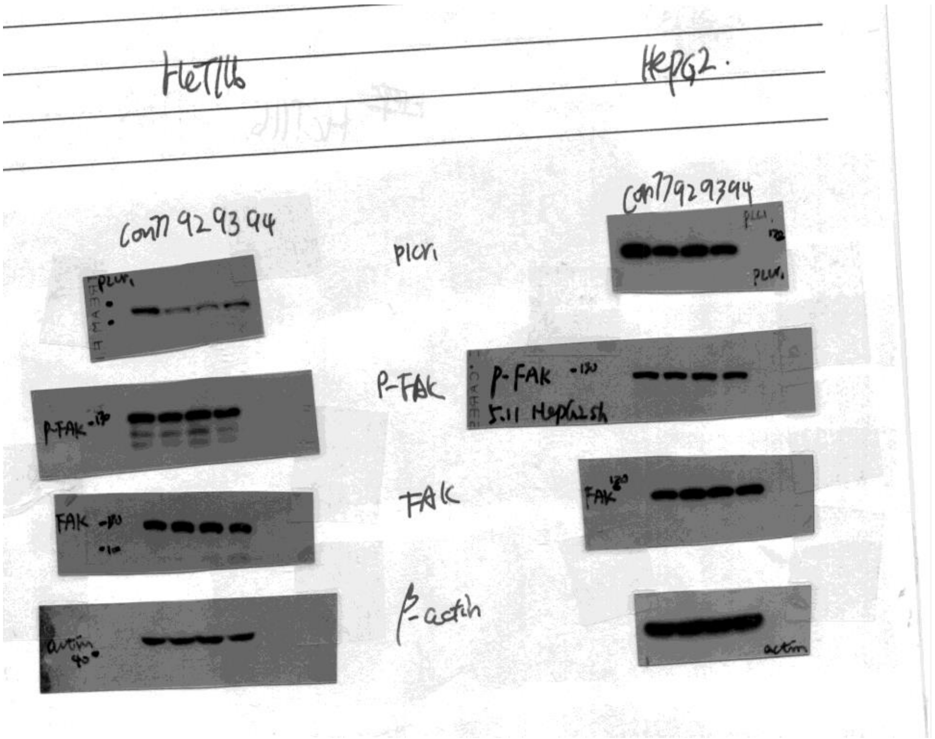

Figure9B(HCT116&HepG2)&Figure10D-1(HepG2)

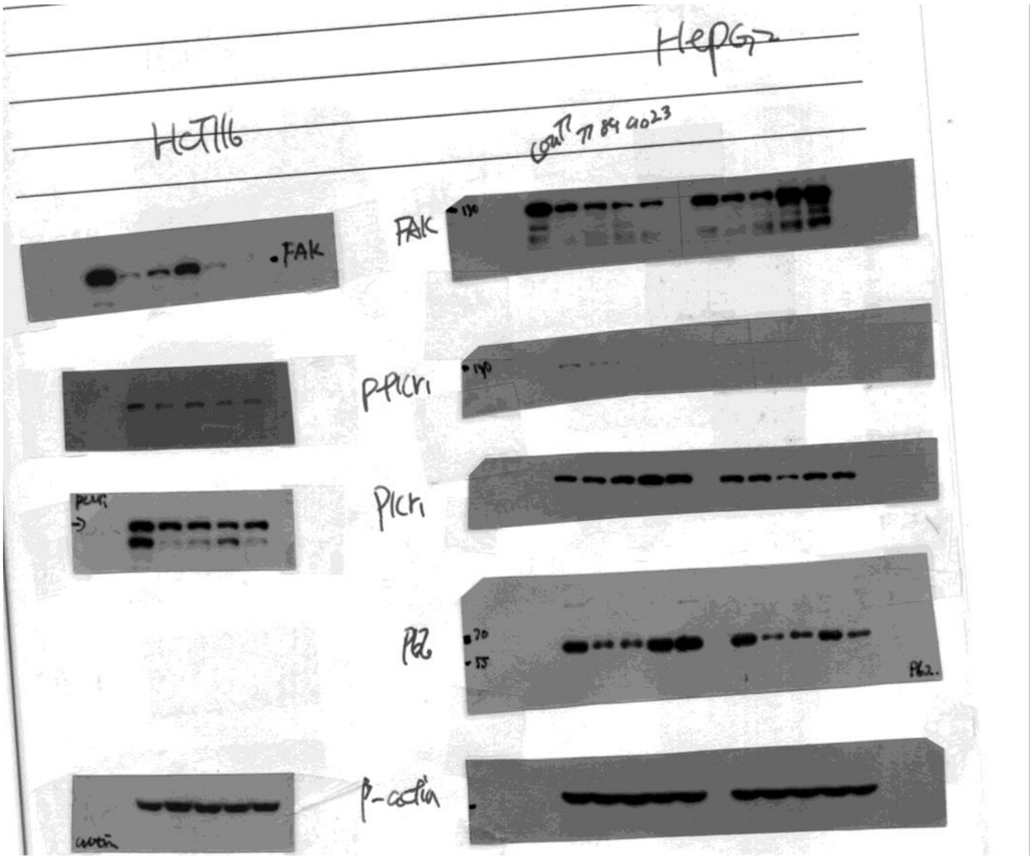

Figure 9C(HCT116&HepG2)

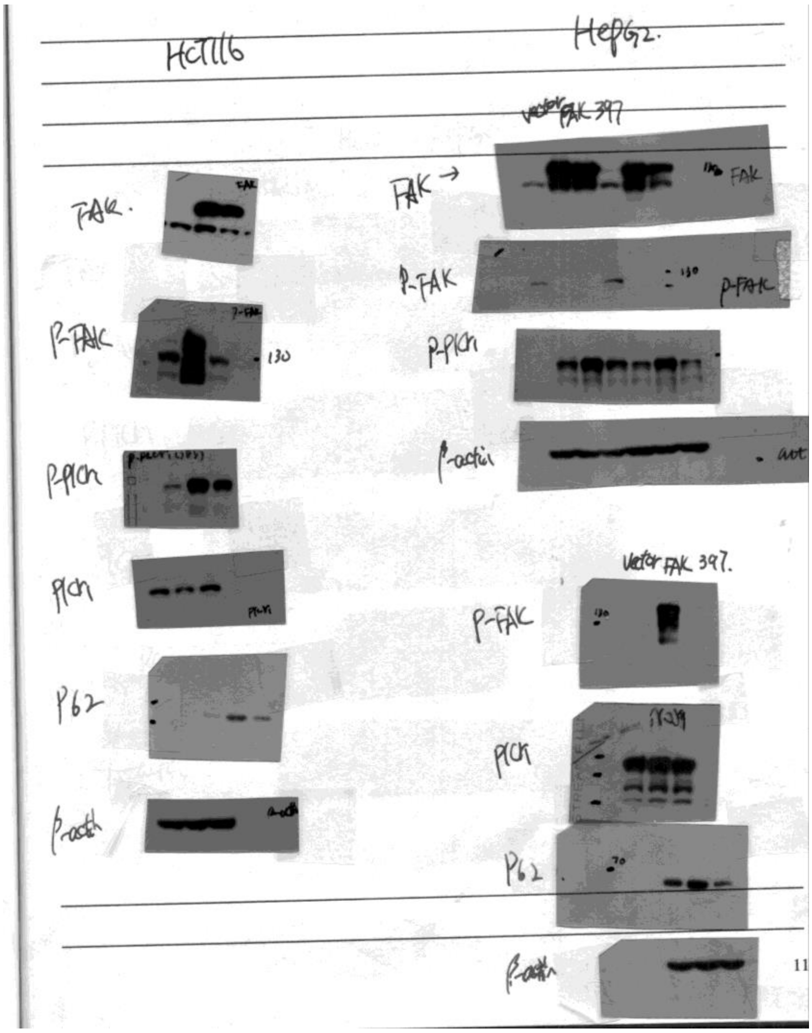

Figure 9D(HCT116&HepG2)

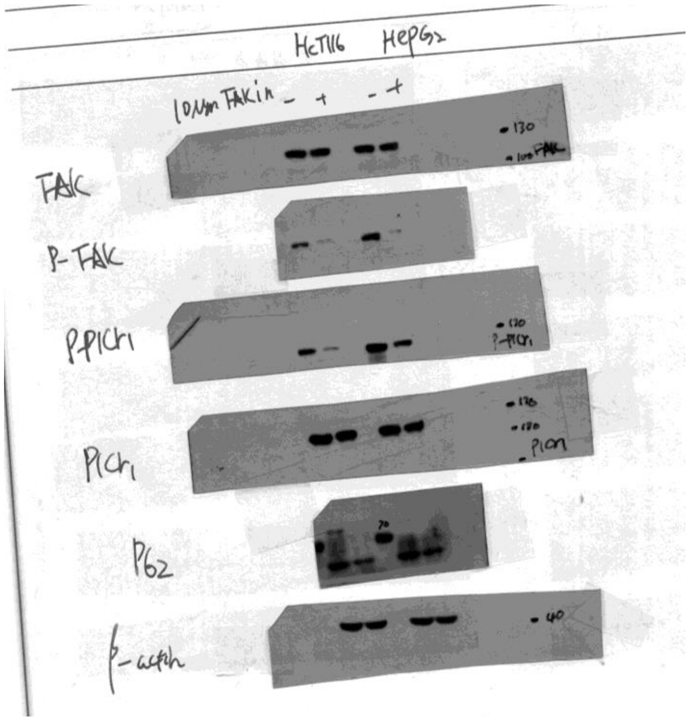

Figure10D-2(HCT116)

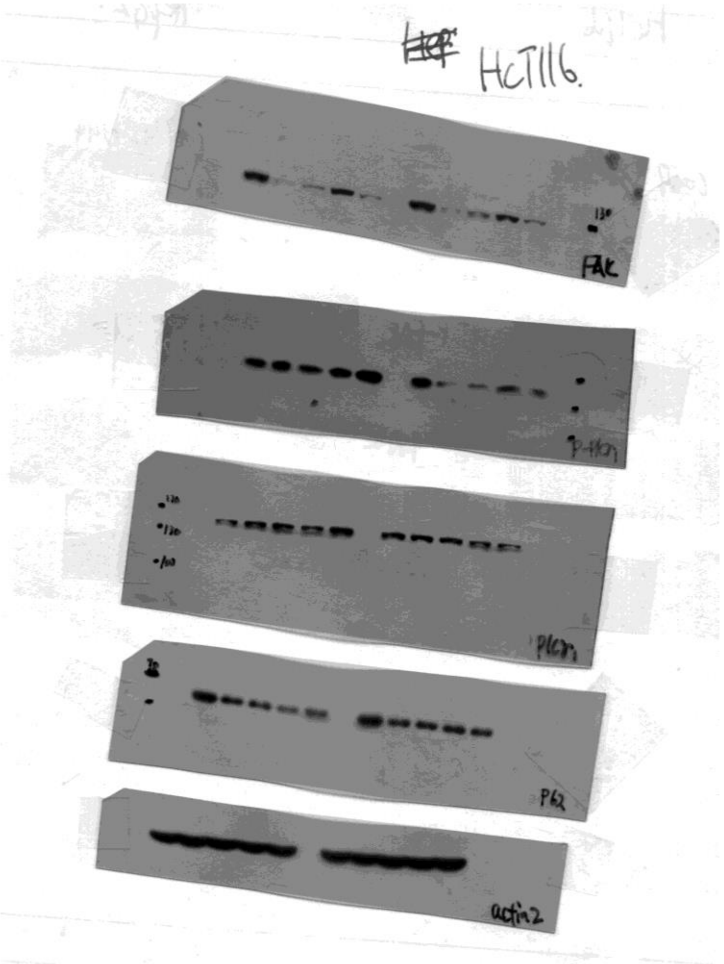

Figure10A(HCT116&HepG2)

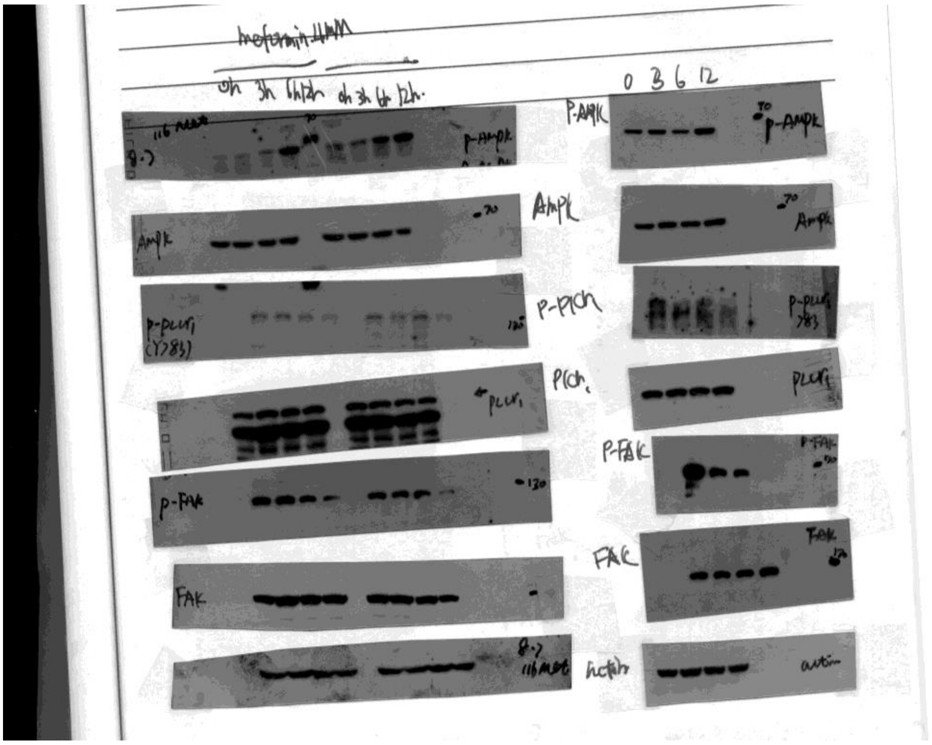

Figure10B

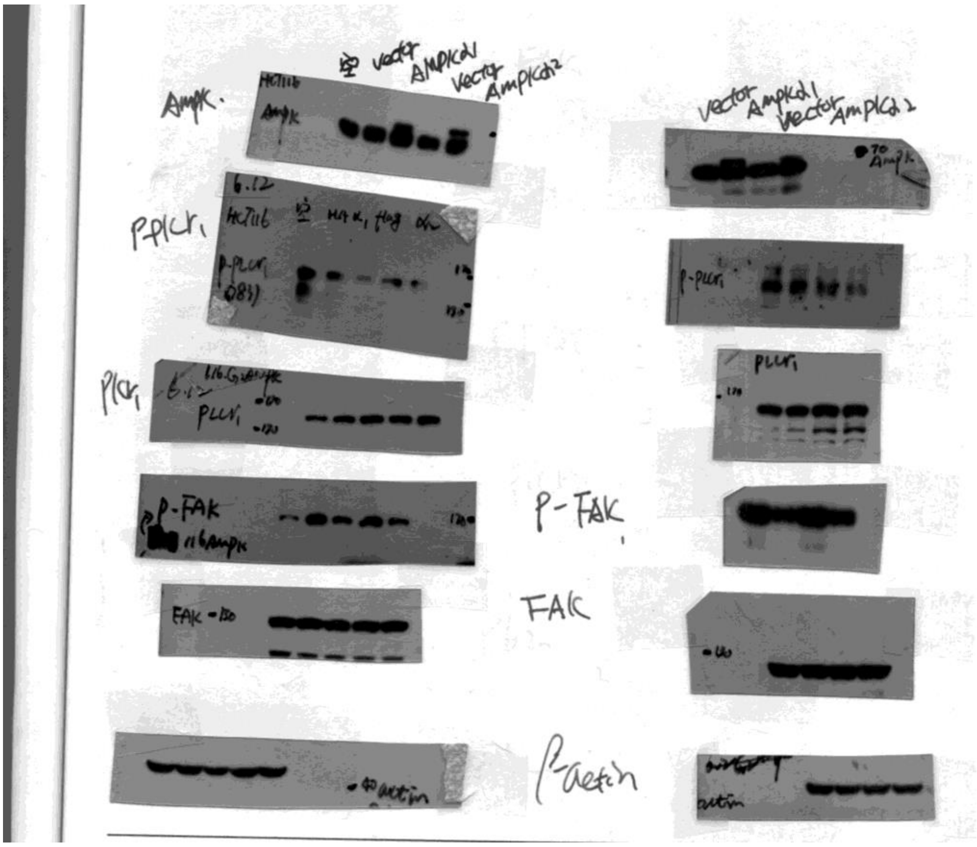

Figure10C(HCT116&HepG2)

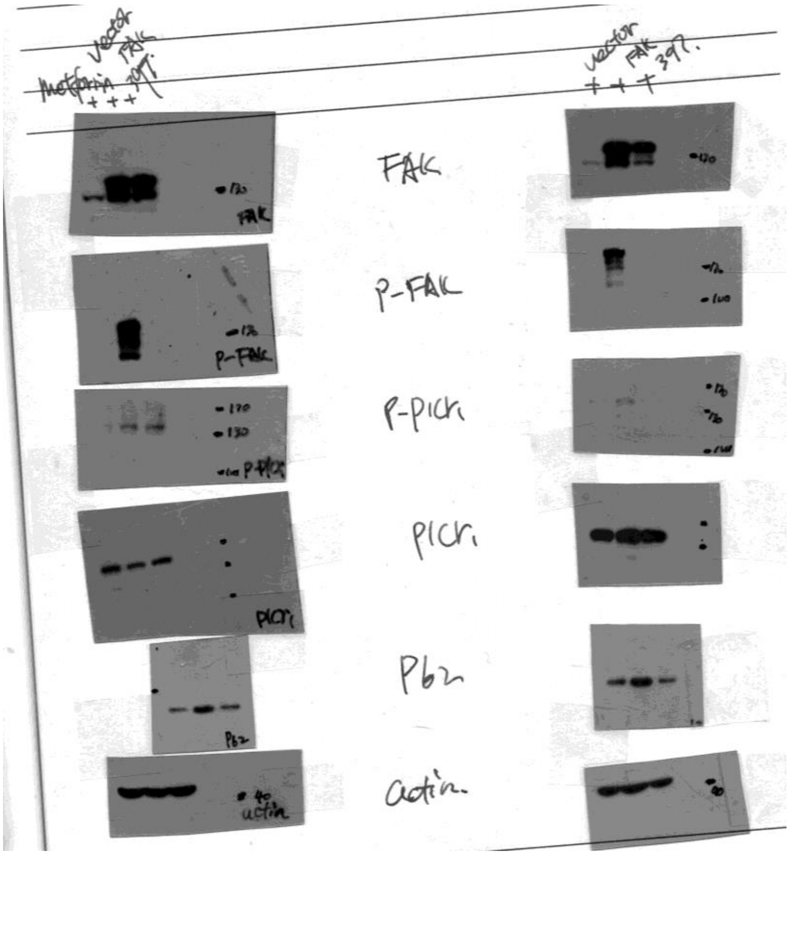

Supplement: Supplementary file 1 — supplementary materials [file 41598_2017_13334_MOESM1_ESM.pdf]
